# Molecular phylogeny and forms of photosynthesis in tribe Salsoleae (Chenopodiaceae)

**DOI:** 10.1093/jxb/erw432

**Published:** 2016-12-21

**Authors:** Christina Schüssler, Helmut Freitag, Nuria Koteyeva, Denise Schmidt, Gerald Edwards, Elena Voznesenskaya, Gudrun Kadereit

**Affiliations:** 1 Botany Department, State Museum of Natural History Stuttgart, Rosenstein, Stuttgart, Germany; 2 Institut für Biologie, Universität Kassel, Heinrich-Plett-Str, Kassel, Germany; 3 Laboratory of Anatomy and Morphology, V. L. Komarov Botanical Institute of Russian Academy of Sciences, St. Petersburg, Russia; 4 School of Biological Sciences, Washington State University, Pullman, WA, USA; 5 Institute für Allgemeine und Spezielle Botanik und Botanischer Garten der Johannes Gutenberg-Universität Mainz, Mainz, Germany; 6 Centre for Organismal Studies Heidelberg, Biodiversity and Plant Systematics, Heidelberg University, Heidelberg, Germany

**Keywords:** Ancestral character state reconstruction, C_2_ pathway, C_3_–C_4_ intermediates, CO_2_ compensation point, leaf anatomy, TEM, western blots

## Abstract

While many C_4_ lineages have Kranz anatomy around individual veins, Salsoleae have evolved the Salsoloid Kranz anatomy where a continuous dual layer of chlorenchyma cells encloses the vascular and water-storage tissue. With the aim of elucidating the evolution of C_4_ photosynthesis in Salsoleae, a broadly sampled molecular phylogeny and anatomical survey was conducted, together with biochemical, microscopic, and physiological analyses of selected photosynthetic types. From analyses of photosynthetic phenotypes, a model for evolution of this form of C_4_ was compared with models for evolution of Kranz anatomy around individual veins. A functionally C_3_ proto-Kranz phenotype (Proto-Kranz Sympegmoid) and intermediates with a photorespiratory pump (Kranz-like Sympegmoid and Kranz-like Salsoloid types) are considered crucial transitional steps towards C_4_ development. The molecular phylogeny provides evidence for C_3_ being the ancestral photosynthetic pathway but there is no phylogenetic evidence for the ancestry of C_3_–C_4_ intermediacy with respect to C_4_ in Salsoleae. Traits considered advantageous in arid conditions, such as annual life form, central sclerenchyma in leaves, and reduction of surface area, evolved repeatedly in Salsoleae. The recurrent evolution of a green stem cortex taking over photosynthesis in C_4_ clades of Salsoleae concurrent with leaf reduction was probably favoured by the higher productivity of the C_4_ cycle.

## Teaser

Evolution of C_3_–C_4_ intermediate and C_4_ lineages are resolved in Salsoleae (Chenopodiaceae), and a model for structural and biochemical changes for the evolution of the Salsoloid form of C_4_ is considered.

## Introduction

Reconstructing the evolution of C_4_ photosynthesis is challenging as it requires the complex coordination of anatomical, ultrastructural, biochemical, and gene regulatory changes from C_3_ ancestors (Hibberd and Covshoff, 2010; Gowik and Westhoff, 2011; Sage *et al.*, 2012, 2014; Williams *et al.*, 2012; Hancock and Edwards, 2014). In bringing together these aspects, a model of C_4_ evolution where Kranz anatomy is formed around individual veins has been developed over the last 30 years, which includes potential evolutionary precursors and a number of transitional, evolutionary-stable states (Monson *et al.*, 1984; Edwards and Ku, 1987; Sage, 2004; Gowik and Westhoff, 2011; Sage *et al.*, 2012, 2014). These hypothetical states are based on distinct phenotypes observed in nature in close relatives of C_4_ lineages and are characterized by a combination of C_3_ and C_4_ characteristics. From these, a stepwise progression from C_3_ to proto-Kranz to photosynthetic intermediates, and finally to C_4_ photosynthesis was proposed with a progressive reduction in photorespiration (Sage *et al.*, 2014; hereafter named the ‘*Flaveria* model’ based on photosynthetic phenotypes studied in this genus).

In dicots, there are many anatomical forms of Kranz anatomy that differ in the arrangement of a dual layer of chlorenchyma cells performing the C_4_ pathway. These includes forms where Kranz anatomy develops around individual veins; however, there are also nine forms where two concentric chlorenchyma layers surround all veins (Edwards and Voznesenskaya, 2011). According to Brown (1975), in C_4_ plants we refer to cells of the inner chlorenchyma layer that become specialized for C_4_ photosynthesis, irrespective of their position in the leaf, as Kranz cells (KC) and the outer layer as mesophyll (M) cells (Edwards and Voznesenskaya, 2011; Voznesenskaya *et al.*, 2013). In C_3_–C_4_ intermediate phenotypes the inner layer of chlorenchyma, which has become specialized to support the C_2_ cycle, is referred to as Kranz-like cells (KLC; Voznesenskaya *et al.*, 2013). In C_3_ species vascular bundles (VB) are surrounded by non-specialized parenchymatic bundle sheath (BS) cells.

Proto-Kranz phenotypes, first described in *Heliotropium* and *Flaveria*, are suggested to represent the initial phase of C_4_ evolution where overall vein density is increased and BS cells have an increased number of organelles, with enlarged mitochondria located internally to chloroplasts in a centripetal position towards the VB (Muhaidat *et al.*, 2011; Sage *et al.*, 2012, 2013, 2014). C_3_–C_4_ intermediate phenotypes, which have been found in grasses and in a number of dicot families, have in common increased development of chloroplasts and mitochondria in the KLCs. Both M and KLC chloroplasts have Rubisco and the C_3_ cycle. In the KLCs there is a distinctive layer of mitochondria that are located internally to the chloroplasts in a centripetal position. In C_3_–C_4_ intermediate phenotypes glycine decarboxylase (GDC) is selectively localized in the KLC mitochondria, which support a C_2_ cycle by establishing a photorespiratory CO_2_ pump. In the C_2_ cycle photorespiratory glycine produced in the M cells is shuttled for decarboxylation by GDC to the KLCs where photorespired CO_2_ is concentrated, enhancing its capture by KLC Rubisco (see Edwards and Ku, 1987; Sage *et al.*, 2012, 2014; Voznesenskaya *et al.*, 2013; Khoshravesh *et al.*, 2016).

For the ‘*Flaveria* model’ C_3_–C_4_ intermediate phenotypes have been classified into two general groups: Type I and Type II C_3_–C_4_ species (Edwards and Ku, 1987; alternatively called Type 1 C_2_ and Type 2 C_2_, Sage *et al.,* 2014). Type I C_3_–C_4_ species have developed little or no capacity for function of a C_4_ cycle as activities/quantities of C_4_ enzymes are low, similar to C_3_ species. These intermediates mainly reduce losses of the CO_2_ generated by photorespiration by its partial refixation in the KLCs. Type II intermediates have substantial expression of a C_4_ cycle; e.g. the levels of the C_4_ cycle enzymes phosphoenolpyruvate carboxylase (PEPC), pyruvate phosphate dikinase (PPDK), and NADP-malic enzyme (NADP-ME) are two- to five-fold higher in Type II C_3_–C_4_ species than in C_3_ species (Ku *et al.*, 1983, 1991; Edwards and Ku, 1987; Moore *et al.*, 1987; Muhaidat *et al.*, 2011; Sage *et al.*, 2012). The values of CO_2_ compensation points (*Γ*) in C_3_–C_4_ intermediate phenotypes are in between those of C_3_ and C_4_ species.

The fact that C_3_–C_4_ intermediate phenotypes thrive, persist, and occasionally have been found in lineages without any C_4_ relatives, suggests that they represent an evolutionary-stable condition in their own right (Monson *et al.*, 1984; Edwards and Ku, 1987). Their predominant occurrence close to C_4_ groups may be strongly biased by the more intensive screening in these lineages. Thus, C_3_–C_4_ intermediate phenotypes do not necessarily represent transitional states that always lead to the establishment of C_4_ photosynthesis. A C_*2*_ cycle might already be favourable in conditions of high photorespiration, e.g. in hot, dry, and saline environments (Keerberg *et al.*, 2014). The ‘*Flaveria* model’ is functionally plausible, and supported by phenotypes that actually exist in nature (Sage *et al.*, 2014); however, phylogenetic evidence for the ancestry of the C_3_–C_4_ intermediate condition is scarce, and is hampered by the generally low number of species with intermediate phenotypes.

C_3_–C_4_ intermediate phenotypes have been recognized in 16 angiosperm genera (Sage *et al.*, 2012, 2014; Khoshravesh *et al.*, 2016). Often, the ancestry of the C_3_–C_4_ intermediate condition is inferred from a sister-group relationship of a C_4_ lineage and a C_3_–C_4_ intermediate lineage (Sage *et al.*, 2011, 2012) because the intermediate condition is *a priori* considered as less derived. However, in such cases it is impossible to distinguish between ancestry and a *de novo* evolution of the C_3_–C_4_ intermediate condition (compare with Hancock and Edwards, 2014). If those cases in which C_3_–C_4_ intermediate photosynthesis seems to precede C_4_ photosynthesis, as suggested in Sage *et al.* (2011; 2012), are critically tested for unequivocal phylogenetic evidence, only *Flaveria* (Asteraceae) studied by McKown *et al.* (2005) holds up. In this case, a stepwise acquisition of C_4_ photosynthesis in one lineage of *Flaveria* was shown (McKown *et al.*, 2005; Lyu *et al.*, 2015).

There are four other promising groups that are rich in C_3_–C_4_ intermediate phenotypes and therefore potentially informative lineages in terms of disentangling the steps of C_4_ evolution and ancestral state reconstruction for C_3_–C_4_ intermediacy: *Blepharis* (Fisher *et al.*, 2015), *Anticharis* (Khoshravesh *et al.*, 2012), *Heliotropium* (Sage *et al.*, 2012), and Salsoleae *sensu stricto* (*s.s.*; Voznesenskaya *et al.*, 2013). A better understanding of C_3_–C_4_ intermediate phenotypes in Salsoleae is particulary important as these, in contrast to the other groups, seem to deviate from the ‘*Flaveria* model’ (see ‘Salsoleae model’, Voznesenskaya *et al.*, 2013).

Salsoleae, especially the former section *Coccosalsola*, has long been known to contain C_3_ and C_4_ species (Winter, 1981; Akhani *et al.*, 1997). In fact, Salsoleae has repeatedly been suspected to contain species that represent reversions from C_4_ back to C_3_ photosynthesis (Carolin *et al.*, 1975; P’yankov *et al.*, 1997; Kadereit *et al.*, 2014); however, this has been questioned by Kadereit *et al.* (2003). According to a survey by Voznesenskaya *et al.* (2013, see table 5) there are at least 21 species with δ^13^C values within the typical range of C_3_ species in Salsoleae. So far, four of these have been shown to possess either proto-Kranz (*Salsola montana*), or a C_3_–C_4_ intermediate phenotype (*S. arbusculiformis*, *S. divaricata*, and *S. laricifolia* (Voznesenskaya *et al.*, 2013 and references therein; Wen and Zhang, 2015). In the ‘*Flaveria* model’ Kranz anatomy is formed around individual veins, requiring a series of anatomical changes in progression from C_3_ to C_4_. In Salsoleae, however, the photosynthetic tissue in leaves forms a continuous layer that surrounds all the vascular and water-storage tissue, i.e. in C_3_ species by multiple layers of mesophyll tissue (Sympegmoid-type anatomy), and in C_4_ species by a dual layer of chlorenchyma tissue forming a Kranz anatomy (Salsoloid-type anatomy). Voznesenskaya *et al.* (2013) proposed a model for transitions from C_3_ to proto-Kranz to C_3_–C_4_ intermediates to C_4_ in Salsoleae, based on limited photosynthetic phenotypes, which would require very different changes in leaf anatomy and regulation of development of the dual layer of chlorenchyma cells compared to that in the ‘*Flaveria* model’ for development of Kranz anatomy around individual veins.

Here, we conduct a large-scale analysis of Salsoleae, including species with C_3_-type δ^13^C values. The results of a molecular phylogenetic study of 74 species and an anatomical survey of 77 species of Salsoleae *s.s*, and some outgroup species, are presented. Furthermore, in a search for additional C_3_–C_4_ intermediates in the tribe, anatomical, ultrastructural, enzyme content, and gas exchange analyses were performed on a number of species that have C_3_-type δ^13^C values. Molecular clock and character optimization analyses were used to reconstruct the evolution of the C_4_ pathway in Salsoleae. The following questions were addressed. (1) Is there evidence for additional C_3_–C_4_ intermediates in Salsoleae? (2) What is the current model for evolution of C_4_ in Salsoleae based on analyses of photosynthetic phenotypes? (3) In what ways does this model differ from the ‘*Flaveria* model’ proposed in Sage *et al.* (2012, 2014)? (4) Where and when did C_4_ photosynthesis originate in Salsoleae, and is there phylogenetic evidence for a reversion from C_4_ back to C_3_? (5) Does the C_3_–C_4_ intermediate condition represent an ancestral state to C_4_ in Salsoleae?

## Material and methods

### Plant material and sampling

Species and samples included in the analyses with their respective voucher information are listed in Supplementary Table S1 at *JXB* online. We used herbarium samples and plants grown in the greenhouse at the Botanical Gardens of the University of Mainz, Germany, and at Washington State University (WSU), Pullman, WA, USA, and leaves of specimens that were fixed during various expeditions, mainly by H. Freitag. A few samples were kindly provided by other institutions. Species of the *Kali* clade were mostly left out, because in the trees based on chloroplast sequence data they are separated from the Salsoleae *s.s.*

In WSU, plants were grown in 15-cm diameter pots with commercial potting soil in a growth chamber (model GC-16; Enconair Ecological Chambers Inc., Winnipeg, Canada) under a 14/10 h 25/18 °C day/night cycle under mid-day PPFD of ~500 μmol quanta m^−2^ s^−1^, and 50% relative humidity for ~2 months. Plants were watered daily and fertilized once per week with Peter’s Professional fertilizer (20:20:20 Scotts Miracle-Gro, Marysville, OH, USA).

Our sampling of Salsoleae for the molecular phylogenetic analyses comprised 74 species representing all currently accepted genera (Supplementary Table S2) and included 15 species with C_3_-like carbon isotope (δ^13^C) values (compare with table 5 in Voznesenskaya *et al.*, 2013). Furthermore, representatives of other primary clades of Salsoloideae (Supplementary Table S2) as well as representatives of Camphorosmoideae were included. For rooting and dating purposes Suaedoideae and Salicornioideae were sampled as outgroups (Supplementary Table S1). For light microscopy 77 species were examined, mostly from the same material (Supplementary Table S1), including 34 species studied for the first time. Data for eight species were taken from the literature. Six species having different anatomical types (most not previously known in that respect) were chosen for study by electron microscopy, and δ^13^C, *in situ* immunolocalization, western blot, and gas exchange analyses.

With respect to nomenclature, apart from a few exceptions we follow previous accounts of the different subfamilies, in particular Botschantzev (1989), Akhani *et al.* (2007), and Kadereit and Freitag (2011), although we are aware that more nomenclatural adjustments are required.

### Sequencing and phylogenetic inference

Total DNA was extracted from dried or fresh leaf material using the DNeasy Plant Mini Kit (QIAGEN, Germany) or innuPREP Plant DNA Kit (Analytik Jena, Germany) following the manufacturers’ protocols but increasing incubation times. PCRs for five markers (*atpB-rbcL* intergenic spacer, *ndhF-rpL32* spacer, *trnQ-rps16* spacer, *rpl16* intron, ITS) were carried out in a T-Professional or T-Gradient Thermocycler (Biometra, Germany), or a PTC100 Thermocycler (MJ Research, USA). Primers sequences, PCR recipes, and cycler programs are documented in Supplementary Table S3. PCR products were checked on 0.8% agarose gels and purified using the NucleoSpin® Gel and PCR clean-up-Kit (Macherey-Nagel, Germany) or ExoSAP (Affymetrix, USA) following the manufacturers’ instructions. The Big Dye® Terminator v3.1 Cycle Sequencing Kit (Applied Biosystems) combined with the primers mentioned above was used for the sequencing reactions, followed by a purification step using IllustraTM SephadexTM G-50 Fine DNA Grade (GE Healthcare, UK). Sequencing was performed following the Sanger method on a 3130xI Genetic Analyzer (Applied Biosystems Inc., USA). The raw forward and reverse sequences were checked and automatically aligned in Sequencher 4.1.4 (Gene Codes Corporation, USA). The refined alignment was performed in Mesquite 2.75 (http://mesquiteproject.org) and carefully checked visually. The program SequenceMatrix (v. 1.7.8; Vaidya *et al.*, 2011) was used to combine the four chloroplast (cp) marker data sets.

Phylogenetic analyses under the settings outlined below were initially conducted individually for the five selected DNA regions. Results of the individual analyses of the four cp markers revealed no topological conflict [i.e. incongruence with ML Bootstrap ≥65% and Posterior Probability (PP) ≥0.90] among individual markers and combination of the cp markers distinctly increased the resolution and support values. For the cp data further analyses were performed using two large and different data sets: (1) a data set with 106 taxa (106t data set) including a broad outgroup sampling; and (2) a data set with 75 taxa (75t data set) in which only *Salsola genistoides* served as the outgroup. The 106t data set was used to reveal primary clades in Salsoloideae and for estimation of divergence time, whereas the 75t data set was used for character optimization (see below). Since the ITS tree and the cp tree showed supported conflict at basal branches, and the combination of cp data and ITS for the 75t data set led to a significant decrease of resolution and support values in some parts of the tree, we concentrated on the cp tree for further analyses and only include the ITS tree for comparison. First, the best-fitting substitution model for the combined cp data sets was inferred using jModelTest (Posada, 2008). CIPRES (Cyberinfrastructure for Phylogenetic Research) Science Gateway V. 3.3. ML phylogenetic analyses were performed using RAxML (Stamatakis, 2006; Stamatakis *et al.*, 2008), including bootstrapping that was halted automatically following the majority-rule ‘autoMRE’ criterion. Bayesian inference (BI) analysis was conducted using BEAST (Bayesian Evolutionary Analysis by Sampling Trees v.1.8.2; Drummond and Rambaut, 2007) with GTR+G (general time-reversible; best-fitting according to jModeltest under AIC criterion) with a gamma-distribution in four categories as the substitution model. A birth-and-death demographic model was used as the tree prior. Markov Chain Monte Carlo (MCMC) analysis was performed with the following settings: randomly generated starting tree, 20 000 000 (106t data set) or 10 000 000 iterations (75t data set), discarded burn-in of 10%, and sampling every 1000 steps (totalling 10 000). For the 106t data set a relaxed clock model was implemented in which rates for each branch are drawn independently from an exponential distribution (Drummond *et al.*, 2006). The crown node of Salsoloideae and Camphoromoideae was set to 47.0–25.5 mya based on divergence-time estimates in the Chenopodiaceae/Amaranthaceae complex (Kadereit *et al.*, 2012). We assumed a uniform distribution within the age bounds set. Other settings were left in default.

### Light and electron microscopy

For light microscopy, after routine checks by manual sectioning, the middle parts of well-developed leaves were selected for transections by a rotary microtome (Leitz 1515). The semi-thin sections of material fixed in FAA [2% (v/v) formaldehyde, 0.5% (v/v) acetic acid, 70% (v/v) ethanol] were studied under a Dialux 20 (Leitz, Wetzlar). Some were first examined and photographed in water to get a better contrast between lignified (blue) and non-lignified (purple) cell walls before embedding into Depex (Serva) for documentation. For detailed study, middle parts of fully developed leaves were fixed and processed in a similar way to that described in Voznesenskaya *et al.* (2013).

For screening purposes, leaf samples from herbarium specimens were first boiled for about 1–3 minutes and hand-cut sections were preserved in glycerol–gelatin. Selected samples for microtome transections were soaked in a 10% solution of NH_3_ for 10 d, dehydrated in ethanol, and embedded in Technovit 7100 (Heraeus Kulzer). The samples were sectioned at 5–20 µm using a rotary microtome. Sections were stained in a 6:6:5:6 mixture of Azur II, Eosin Y, methylene blue, and distilled water and mounted in Eukitt (O. Kindler) after drying. Images of the sections were taken using a Leitz Diaplan light microscope combined with Leica Application Suite 2.8.1.

For ultrastructural characterization, ultra-thin sections were taken from the same samples prepared for the light microscopy study and embedded in Spurr’s resin as described in Voznesenskaya *et al.* (2013). The number and sizes of mitochondria in chlorenchyma cells were estimated per cell section (about 10–15 cell images from 2–3 separate leaf samples) using an image analysis program (ImageJ 1.37v, https://imagej.nih.gov/ij/index.html).

### δ^13^C values

Carbon isotope composition of plant samples was determined at Washington State University using a standard procedure relative to PDB (Pee Dee Belemnite) limestone as the carbon isotope standard (Bender *et al.*, 1973). Leaf samples (from plants growing in the WSU growth chamber) were dried at 60 °C for 24 h, and then 1–2 mg were placed in a tin capsule and combusted in a Eurovector elemental analyser. The resulting N_2_ and CO_2_ gases were separated by gas chromatography and admitted into the inlet of a Micromass Isoprime isotope ratio mass spectrometer (IRMS) for determination of ^13^C/^12^C ratios (*R*). δ^13^C values were determined where δ = 1000 × (*R*_sample_/*R*_standard_) − 1.

### *In situ* immunolocalization

Sample preparation and immunolocalization by transmission electron microscopy (TEM) were carried out according to Voznesenskaya *et al.* (2013). The antibody used (raised in rabbit) was against the P subunit of glycine decarboxylase (GDC) from *Pisum sativum* L. (courtesy of D. Oliver, Iowa State University). Pre-immune serum was used for controls. The density of labeling was determined by counting the gold particles on electron micrographs and calculating the number per unit area (µm^2^) using ImageJ 1.37v. For each cell type, replicate measurements were made on parts of cell sections (*n* = 10–15 cell images). Immunolabeling procedures were performed separately for different species; the difference in the labeling intensity reflects the difference between cell types but not between species. The level of background labeling was low in all cases.

### Western blot analysis

Extraction of total soluble proteins, protein separation, and blotting onto a nitrocellulose membrane were carried out according to Voznesenskaya *et al.* (2013). A loading control with protein samples (20 µg) separated by 10% (w/v) SDS-PAGE can be found in Supplementary Fig. S1. Western blots were performed using anti-*Amaranthus hypochondriacus* NAD-malic enzyme (NAD-ME) IgG, which was prepared against the 65-kDa α subunit (courtesy of J. Berry; Long and Berry, 1996) (1:5000), anti-*Zea mays* 62-kDa NADP-malic enzyme (NADP-ME) IgG (courtesy of C. Andreo; Maurino *et al.*, 1996) (1:5000), anti-*Zea mays* PEPC IgG (1:100 000), and anti-*Zea mays* pyruvate,Pi dikinase (PPDK) IgG (courtesy of T. Sugiyama) (1:5000). The intensities of bands in western blots were quantified using ImageJ 1.37v and expressed relative to the level in the C_4_ species *S. oppositifolia*, which was set at 100%.

### CO_2_ compensation point

Measurements of CO_2_ compensation points (*Γ*) were made on an individual lateral branch using a Li-Cor lighted chamber (LI-6400-22L; Li-Cor Biosciences, Lincoln, NE, USA) designed for terete or semi-terete conifer leaves. For each species, a part of a branch of an intact plant was enclosed in the chamber and illuminated with a PPFD of 1000 μmol quanta m^−2^ s^−1^ under 400 μmol mol^−1^ CO_2_ at 25 ºC until a steady-state rate of CO_2_ fixation was obtained (generally 45–60 min). For varying CO_2_ experiments, the CO_2_ level was first decreased, and then increased up to 400 μmol mol^−1^ at 5 min intervals. *Γ* was determined by extrapolation of the initial slope of rate of CO_2_ fixation (*A*) versus the intercellular CO_2_ concentration in the leaf (*C*_i_) through the *x*-axis where the net rate of CO_2_ assimilation equals zero. The leaf area exposed to the incident light was calculated by taking a digital image of the part of the branch that was enclosed in the chamber, and then determining the exposed leaf area using ImageJ 1.37v.

### Statistical analysis

Where indicated, standard errors were determined, and ANOVA was performed using Statistica 7.0 software (StatSoft, Inc.). Tukey’s HSD (honest significant difference) test was used to analyze differences between amounts of gold particles in BS/KLC/KC versus M for each species, and δ^13^C and *Г* values in different species. All analyses were performed at the 95% significance level.

### Character coding and analyses of character evolution

Analyses of character evolution were conducted for five traits: (1) type of photosynthesis; (2) KC/KLC function; (3) life form; (4) leaf sclerenchyma; and (5) leaf reduction (Table 1). Traits were optimized over 1000 trees of 74 Salsoleae and *Salsola genistoides* as the outgroup obtained in a Bayesian analysis (see above) using the ML criterion in Mesquite (http://mesquiteproject.org). The fit of single- versus two-rate models was tested for traits with two character states using a likelihood ratio test. Table 1 gives information about the coding of the character states of the five traits.

**Table 1.** Traits of photosynthetic pathway, leaf anatomy and life form in Salsoleae s.s. **Trait 1: type of photosynthesis according to carbon isotope value; coding trait 2:** C_3_ = 0, C_4_ = 1. **Trait 2: function of bundle sheath (BS), Kranz-like (KLC) and Kranz cells (KC); coding trait 2:** C_3_ type BS cells around peripheral VB (few or no organelles) = 0, KLC with increased number of organelles mostly in centripetal position, GDC only expressed in KLC cells, C_3_-C_4_ species = 1, C_4_ type Kranz cells = 2. **Trait 3: Life form** according to standard floras and our own observations in the field; **coding trait 3:** perennial l = 0, annual = 1. **Trait 4: presence of sclerenchyma** by replacement of major parts of the central water storage tissue; **coding trait 4:** no = 0, yes = 1. **Trait 5: sites of major photosynthetic function; coding trait 5:** predominantly in leaves and leaf-like structures (0), ± equally in leaves and stems (1), predominantly in stems due to reduction of leaves or their trans-formation into thorns (2). References for photosynthetic pathway and leaf anatomy see below table. Species are classified according to carbon isotope composition of leaf tissue as C_3_ or C_4_. Species are classified as C_3_, proto-kranz, C_3_-C_4_ intermediates, and C_4_ based on analyses of leaf anatomy, Trait 2 and C isotope composition, (see text).

Species of Salsoleae *s.s.*	Isolate no. for molecular analysis	Trait 1: Type of photosynthesis according to carbon isotope ratio	Leaf anatomy; type names according to Voznesenskaya *et al.* (2013)	Trait 2	Trait 3	Trait 4	Trait 5
*Anabasis aphylla* L.	chen 2743/2017	C_4_ (1, 2, 12)	salsoloid+H (1, 6, 8, this study)	2	0	0	2
*Anabasis articulata* (Forssk.) Moq.	chen 2360	C_4_ (7)	salsoloid+H (6, 8, 12, this study)	2	0	0	2
*Anabasis brevifolia* C.A. Mey.	chen 2407	C_4_ (12)	salsoloid+H (2, 11, this study)	2	0	0	1
*Anabasis calcarea* (Charif & Aellen) Bokhari & Wendelbo	chen 1841	C_4_ (1)	salsoloid+H (1, this study)	2	0	0	2
*Anabasis ehrenbergii* Schweinf. ex Boiss.	chen 2403/2741	C_4_ (12)	salsoloid+H (this study)	2	0	0	2
*Anabasis setifera* Moq.	chen 2373	C_4_ (7)	salsoloid+H (1, 2, 6, 12, this study)	2	0	0	1
*Arthrophytum betpakdalense* Korov.	chen 0229	C_4_ **	salsoloid+H (this study)	2	0	0	1
*Arthrophytum gracile* Aellen	chen 2603	C_4_ (1)	salsoloid+H (this study)	2	0	0	2
*Arthrophytum lehmannianum* Bunge	chen 2637	C_4_ (4)	salsoloid+H (5, this study)	2	0	0	1
*Cornulaca amblyacantha* Bunge	chen 0350	C_4_ **	salsoloid+H+S (this study)	2	0	1	1
*Cornulaca monacantha* Delile	chen 0212	C_4_ (1, 12)	salsoloid+H+S (2, this study)	2	0	1	1
*Cornulaca setifera* (DC.) Moq	chen 0304	C_4_ (12)	salsoloid+H+S (6, this study)	2	0	1	1
*Cyathobasis fruticulosa* (Bunge) Aellen	chen 0082	C_4_ (12)	salsoloid+H+S (this study)	2	0	1	2
*Girgensohnia diptera* Bunge	chen 2639	C_4_ **	salsoloid+H+S (this study)	2	1	1	2
*Girgensohnia minima* E. Korov.	chen 2601	C_4_ **	salsoloid+H+S (this study)	2	1	1	2
*Girgensohnia oppositiflora* (Pall.) Fenzl	chen 0033	C_4_ (1, 12)	salsoloid+H+S (2, 6, this study)	2	1	1	(1)2
*Gyroptera gillettii* Botsch	chen 2819	C_4_ **	salsoloid+H (this study)	2	0	0	0
*Halogeton alopecuroides* (Delile) Moq.	chen 0300	C_4_ (1, 7, 12)	salsoloid+H (6, 8, 12, this study)	2	0	1	0
*Halogeton arachnoideus* Moq.	chen 2605	C_4_ (1)	salsoloid+H (this study)	2	1	0	0
*Halogeton glomeratus* (M. Bieb.) C.A. Mey.	chen 0030	C_4_ (1)	salsoloid+H (2)	2	1	0	0
*Halogeton sativus* (L.) Moq.	chen 1229	C_4_ (1)	salsoloid+H (8, this study)	2	1	0	0
*Halothamnus bottae* Jaub. & Spach	chen 0351	C_4_ (12)	salsoloid (7)	2	0	0	2
*Halothamnus ferganensis* Botsch.	chen 0197	C_4_ **	salsoloid (7)	2	0	0	1
*Halothamnus iliensis* (Lipsky) Botsch.	chen 2668	C_4_ (1, 12)	salsoloid (7)	2	1	0	1
*Halothamnus somalensis* (N.E. Br.) Botsch.	chen 2584	C_4_ **	salsoloid (7)	2	0	0	2
*Haloxylon ammodendron* (C.A. Mey.) Bunge	chen 0035	C_4_ (1, 2, 12)	salsoloid+H (11, this study)	2	0	0	2
*Haloxylon persicum* Bunge ex Boiss.	chen 2815	C_4_ (7, 12)	salsoloid+H (11)	2	0	0	2
*Hammada articulata* (Moq.) O. Bolos & Vigo	chen 0196	C_4_ **	salsoloid+H (2, 6, this study))	2	0	0	2
*Hammada eriantha* Botsch.	chen 2813	C_4_ **	salsoloid+H (this study)	2	0	0	2
*Hammada griffithii* (Moq.) Iljin	chen 2635	C_4_ (1, 12)	salsoloid+H+S (this study)	2	0	1	2
*Hammada negevensis* Iljin & Zoh.	chen 2814	C_4_ (7, 12)	salsoloid+H (this study)	2	0	0	2
*Hammada salicornica* (Moq.) Iljin	chen 2752	C_4_ (1, 7)	salsoloid+H (2, 8, 12, this study)	2	0	0	2
*Hammada schmittiana* (Pomel) Botsch.	chen 2629	C_4_ (7, 12)	salsoloid+H (this study)	2	0	0	2
*Hammada thomsonii* (Bunge) Iljin	chen 0178	C_4_ **	salsoloid+H+S (this study)	2	0	1	2
*Horaninowia capitata* Sukhor.	chen 0188	C_4_ **	salsoloid+H+S (this study)	2	1	1	1
*Horaninowia platyptera* Charif & Aellen	chen 2602	C_4_ (1)	salsoloid+H+S (this study)	2	1	1	1
*Horaninowia ulicina* Fisch. & C.A. Mey.	chen 2589	C_4_ (1)	salsoloid+H+S (15, this study)	2	1	1	1
*Iljinia regelii* (Bunge) Korovin	chen 0182	C_4_ (4)	salsoloid+H (11, this study)	2	0	0	0
*Lagenantha cycloptera* (Stapf) M.G. Gilbert & Friis	chen 2809	C_4_ **	salsoloid+H (this study)	2	0	0	0
*Noaea minuta* Boiss. & Bal.	chen 0079	C_4_ (12)	salsoloid+S (this study)	2	1	1	0
*Noaea mucronata* (Forssk.) Asch. & Schweinf.	chen 0019	C_4_ (1, 2, 7)	salsoloid+S (6, 9, this study)	2	0	1	(1)2
*Nucularia perrinii* Batt.	chen 2627	C_4_ **	salsoloid+H (8, this study)	2	0	0	0
*Rhaphidophyton regelii* (Bunge) Iljin	chen 0075	C_3_ (4)	kranz-like salsoloid+S (this study)	1	0	1	0
*Salsola abrotanoides* Bunge	chen 2996	C_3_ (4, 6, 9)	sympegmoid (11, this study)	0	0	0	0
*Salsola acutifolia* (Bunge) Botsch.	chen 2640	C_4_ (1, 12)	salsoloid+H (this study)	2	1	0	0
*Salsola arbusculiformis* Drob.	chen 0176	C_3_ (1, 2, 6, 8, 11)	kranz-like sympegmoid (13, 16, this study)	1	0	0	0
*Salsola botschantzevii* Kurbanov	chen 2630	C_3_ (9)	proto-kranz sympegmoid (this study)	0	0	0	0
*Salsola cyrenaica* (Maire & Weiller) Brullo	chen 0354	C_4_ (9)	salsoloid+H (3, this study)	2	0	0	0
*Salsola deschaseauxiana* Litard. & Maire	chen 2758 ( = 2641)	C_3_ (9)	kranz-like salsoloid (this study)	1	0	0	0
*Salsola divaricata* Masson ex Link	chen 2779	C_3_ (6, 9)	kranz-like salsoloid (14, this study)	1	0	0	0
*Salsola drobovii* Botsch.	chen 0175	C_3_ (4, 6, 9)	proto-kranz sympegmoid (this study)***	0	0	0	0
*Salsola florida* (M. Bieb.) Poir.	chen 2811	C_4_ (1, 12)	salsoloid+H (2, 6, this study)	2	1	0	0
*Salsola foliosa* (L.) Schrad.	chen 0103	C_4_ (1)	salsoloid+H (11, this study)	2	1	0	0
*Salsola grandis* Freitag, Vural & N. Adιgüzel	chen 0105	C_4_ **	salsoloid+H (4, this study)	2	1	0	0
*Salsola gymnomaschala* Maire	chen 0355	C_3_ (9)	kranz-like salsoloid (this study)	1	0	0	0
*Salsola junatovii* Botsch.	not included	C_3_ (9)	proto-kranz sympegmoid (this study)	0	0	0	0
*Salsola kerneri* (Woł.) Botsch.	chen 2642	C_4_ (1, 6)	salsoloid+H (this study)	2	0	0	0
*Salsola laricifolia* Turcz. ex Litv.	chen 1355	C_3_ (6, 9, 10, 11)	kranz-like salsoloid (14, 15, 16, this study)	1	0	0	0
*Salsola lipschitzii* Botsch.	not included	C_3_ (9)	proto-kranz sympegmoid (this study)	0	0	0	0
*Salsola melitensis* Botsch.	chen 2644	C_4_ (9)	salsoloid+H (this study)	2	0	0	0
*Salsola montana* Litv.	chen 2591	C_3_ (1, 2, 5, 6, 9)	proto-kranz sympegmoid (14)	0	0	0	0
*Salsola oppositifolia* Desf.	chen 0099	C_4_ (1, 6, 9, 12)	salsoloid+H (8, this study)	2	0	0	0
*Salsola oreophila* Botsch.	chen 2847	C_3_ (5, 9)	sympegmoid (10, this study)	0	0	0	0
*Salsola pachyphylla* Botsch.	chen 2762	C_3_ (5, 6)	sympegmoid (11, this study))	0	0	0	0
*Salsola rosmarinus* (Ehrenb. ex Boiss.) Akhani	chen 0303	C_4_ (1, 7)	salsoloid+H (2, 6, this study)	2	0	0	0
*Salsola schweinfurthii* Solms-Laub.	chen 2827	C_4_ (1, 6, 7, 12)	salsoloid+H (this study)	2	0	0	0
*Salsola soda* L.	chen 2834	C_4_ (1, 7)	salsoloid+H (9, this study)	2	1	0	0
*Salsola stocksii* Boiss.	chen 2646	C_4_ (1)	salsoloid+H (2)	2	0	0	1
*Salsola tianschanica* Botsch.	not included	C_3_ (9)	sympegmoid (this study)	0	0	0	0
*Salsola tunetana* Brullo	chen 2647	C_4_ **	salsoloid+H (this study)	2	0	0	0
*Salsola verticillata* Schousboe	chen 2648	C_3_ (this study)*	kranz-like salsoloid (this study)	1	0	0	0
*Salsola webbii* Moq.	chen 2828	C_3_ (1, 6, 9, 12)	sympegmoid (2, 8, 14, this study)	0	0	0	0
*Salsola zygophylla* Batt. & Trab.	chen 2756	C_4_ (1, 6, 12)	salsoloid+H (this study)	2	0	0	0
*Salsola zygophylloides* (Aellen & Townsend) Akhani	chen 2593	C_4_ **	salsoloid+H (this study)	2	0	0	0
*Sevada schimperi* Moq.	chen 2590	C_4_ (3)	salsoloid+H (this study)	2	0	0	0
*Sympegma regelii* Bunge	chen 383a/2766	C_3_ (4, 11)	sympegmoid (2, 9, 16, this study)	0	0	0	0
*Salsola genistoides* Juss. ex Poir. (outgroup)	chen 1155/1362	C_3_ (1, 9)	sympegmoid (11, this study)	0	0	0	2

**References for C**_**3**_ **versus C**_**4**_ **type carbon isotope ratio:** 1 = Akhani *et al.* (1997), 2 = Akhani and Ghasemkhani (2007), 3 = Carolin *et al.* (1975), 4 = Freitag and Stichler (2000), 5 = P’yankov *et al.* (1997), 6 = Pyankov *et al.* (2001), 7 = Shomer-Ilan *et al.* (1981), 8 = Voznesenskaya *et al.* (2001), 9 = Voznesenskaya *et al.* (2013), 10 = Wen and Zhang (2011), 11 = Wen and Zhang (2015), 12 = Winter (1981).

**References for leaf anatomy:** 1 = Bokhari and Wendelbo (1978), 2 = Carolin *et al.* (1975), 3 = Freitag and Duman (2000), 4 = Freitag *et al.* (1999), 5 = Freitag and Stichler (2000), 6 = Khatib (1959), 7 = Kothe-Heinrich (1993), 8 = Maire (1962), 9 = Monteil (1906), 10 = P’yankov *et al.* (1997), 11 = Pyankov *et al.* (2001), 12 = Volkens (1887), 13 = Voznesenskaya *et al.* (2001), 14 = Voznesenskaya *et al.* (2013), 15 = Wen and Zhang (2011), 16 = Wen and Zhang (2015).

*In Voznesenskaya *et al.* (2013) this species was mentioned to have the C_4_ pathway. However, samples for the carbon isotope value were taken from a wrongly identified specimen [D. Podlech 44954 (P)]. The correct identification for this specimen is *Salsola oppositifolia*, which indeed is a C_4_ species.

** C_4_ metabolism deduced from leaf anatomy, no carbon isotope values available.

*** Classified as sympegmoid in Freitag and Duman (2000), Pyankov *et al.* (2001) and Khatib (1959).

## Results

### Leaf anatomy

#### Light microscopy

**Fig. 1.**
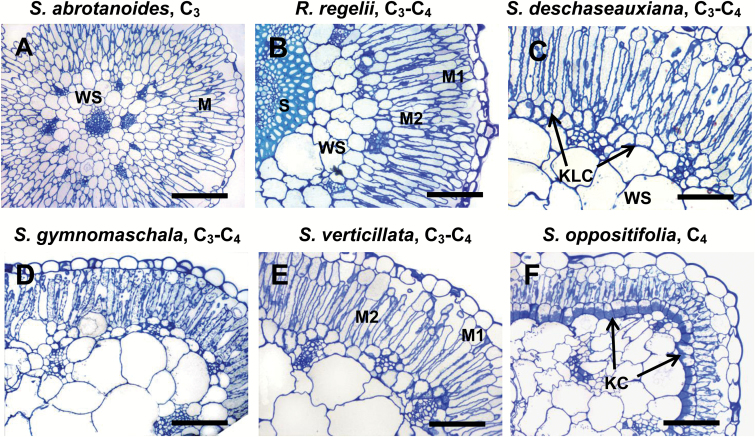
General anatomy in leaves of five *Salsola* species (A, C–F) and *Rhaphidophyton regelii* (B). *Salsola abrotanoides* (A), *S. deschaseauxiana* (C), *S. gymnomaschala* (D), *S. verticillata* (E), and *S. oppositifolia* (F). The images show light microscopy on leaf cross-sections illustrating the position of the palisade mesophyll (M) and bundle sheath (BS)/Kranz-like cells (KLCs)/Kranz cells (KCs). Note the continuous layer of KLCs in *R. regelii*, *S. deschaseauxiana*, *S. gymnomaschala*, and *S. verticillata*, and the difference between the outer (M1) and inner (M2) layers of mesophyll. Sclerenchyma (S) and water-storage (WS) tissue are also indicated. Scale bars = 200 μm for (A); 100 μm for (B–F).

Figure 1A shows that *S. abrotanoides* (C_3_) mostly had two layers of palisade M cells. The peripheral vascular bundles (VBs) were surrounded by a layer of bundle sheath (BS) cells, which looked similar to the adjacent cells of the water-storage (WS) tissue but were smaller.

**Fig. 2.**
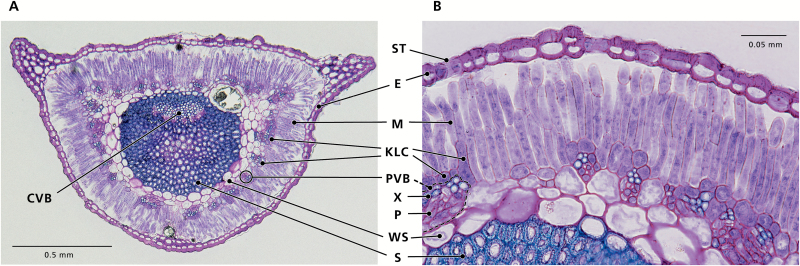
Leaf cross-sections of *Rhaphidophyton regelii*, a C_3_–C_4_ species with Kranz-like Salsoloid leaf anatomy. (A) Cross-section of entire leaf, and (B) close-up of the chlorenchyma. Abbreviations: E, epidermis; M, mesophyll; KLC, Kranz-like cells; WS, water storage tissue; S, sclerenchyma; CVB, central vascular bundle; PVB, peripheral vascular bundles; X, xylem; P, phloem; ST, stoma.

Four species (all C_3_–C_4_), *R. regelii*, *S. deschaseauxiana*, *S. gymnomaschala*, and *S. verticillata* (Fig. 1B–E) were similar in having two layers of chlorenchyma cells underneath the epidermis. While the inner layer consisted of elongated palisade cells (M2), the cells of the outer layer (M1) were 2–5 times shorter with a reduction to varying degrees in the number of chloroplasts depending on species and growth conditions. In *R. regelii* the M1 cells were elongated (Figs 1B and 2), but in the other species the M1 cells appeared almost globular to polyhedral in shape; they were wider than the M2 palisade cells and more similar to the typical hypodermis of C_4_ species (Fig. 1C–E). These species had a clearly defined continuous (or almost continuous in *R. regelii*) layer of chlorenchymatous Kranz-like cells (KLCs), which was situated above and between the peripheral VBs.

In *S. oppositifolia* (C_4_) the palisade M2 cells were rather short and M1 cells were represented by a typical hypodermis consisting of large globular cells that were almost devoid of chloroplasts. The KCs had organelles in a centripetal position; they formed a continuous layer just beneath the palisade cells (Fig. 1F).

Among the *Salsola* species in this study, C_3_*S. abrotanoides* had the lowest volume of WS tissue. In *R. regelii*, the inner part of the WS tissue was replaced by massive sclerenchyma tissue, which accounted for half of the leaf diameter and for the stiff appearance of the leaves (Fig. 2). Crystal-bearing idioblasts were preferentially located in the hypodermis or hypodermis-like layer, and in the Kranz-like layer between the peripheral bundles, but they could occur scattered elsewhere. In *S. gymnomaschala* and in *S. verticillata* the epidermis was partially doubled. In all *Salsola* species the main vein was located more or less in the center of the leaf and surrounded by 2-4 layers of WS tissue.

#### Transmission electron microscopy

**Fig. 3.**
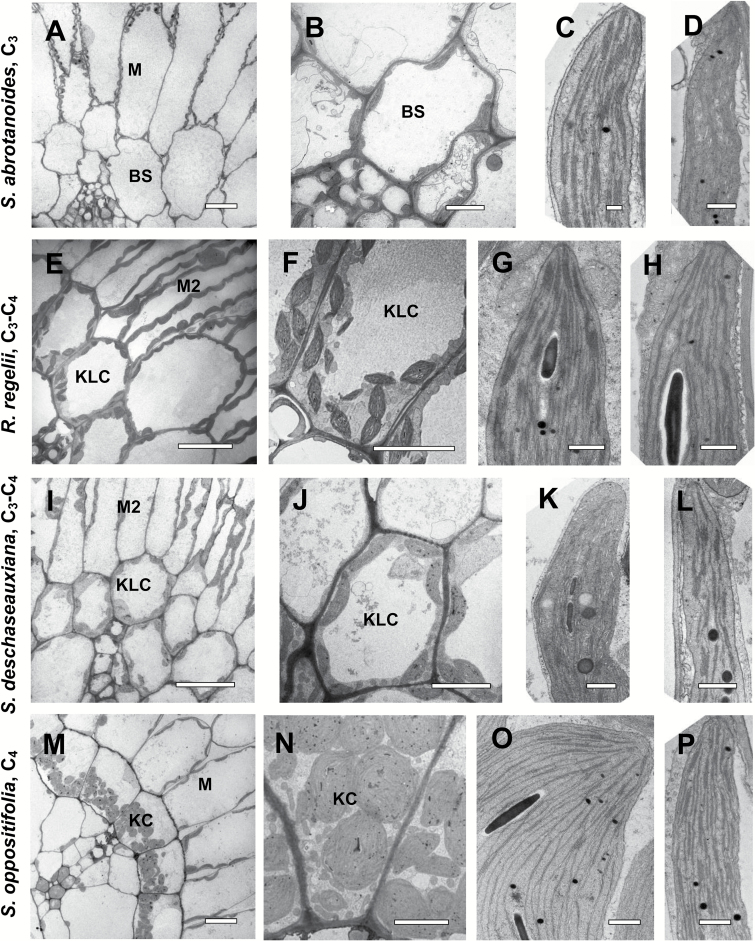
Electron microscopy of bundle sheath (BS)/Kranz-like cells (KLCs)/Kranz cells (KCs) and mesophyll (M) chlorenchyma cells in leaves of three *Salsola* species and *Rhaphidophyton regelii*: *S. abrotanoides* (A–D), *R. regelii* (E–H), *S. deschaseauxiana* (I–L), and *S. oppositifolia* (M–P). (A, E, I, M) Micrographs show M and BS/KLC/KC around vascular bundles. (B, F, J, N) Organelle distribution in BS/KLC/KC at a higher magnification. Note the difference in abundance of organelles in BS/KLC/KC between species, and the numerous mitochondria in KLCs of *R. regelii* (F) and *S. deschaseauxiana* (J), and in KCs in *S. oppositifolia* (N). (C, G, K, O) Chloroplast structure in BS/KLC/KC. (D, H, L, P) Structure of M chloroplasts. Scale bars = 20 μm for (A, I, M); 10 μm for (E,J, M); 5 μm for (N); 1 μm for (F); 0.5 μm for (B–D, G, H, K, L, O, P).

Figure 3 shows obvious differences in the quantity, position, size, and level of development of BS cell organelles in the C_3_ species and in the corresponding KLCs in intermediates and the KCs of the C_4_ species.

C_3_*Salsola abrotanoides* (Fig. 3A, B) had the lowest number of organelles in BS cells; a few chloroplasts and mitochondria were distributed more or less evenly along the cell wall, with some mitochondria located in a centrifugal position. The structure of the thylakoid system was similar for BS and M chloroplasts (Fig. 3C, D).

In the KLCs of four species identified as C_3_–C_4_ intermediates, *R. regelii* (Fig. 3E, F), *S. deschaseauxiana* (Fig. 3I, J), *S. gymnomaschala*, and *S. verticillata* (not shown), the chloroplasts were at least twice as numerous (per cell section) than those of the BS in *S. abrotanoides*; they were distributed along the cell wall but tended to be enriched in the centripetal position. The mitochondria were also twice as numerous (per cell section) and 1.5–2 times larger than in BS cells of *S. abrotanoides*; and most of them were located in the centripetal position, close to the inner periclinal or radial cell walls (Fig. 3F, J). KLC chloroplasts (Fig. 3G, K) and M chloroplasts (Fig. 3H, L) in *R. regelii* and *S. deschaseauxiana* (and the other two *Salsola* intermediates, not shown) had a similar structure with a well-developed system of medium-sized grana consisting of 7–11 thylakoids.

The KCs in C_4_*S. oppositifolia* contained numerous organelles in the centripetal position (Fig. 3M, N). The chloroplast structure differed remarkably among M cells and KCs: while the M chloroplasts had small to medium-sized grana of 2–5 thylakoids in stacks (Fig. 3P), the KC chloroplasts had numerous single thylakoids that interconnect small grana of paired thylakoids, or a few grana consisting mostly of 3–5 thylakoids (Fig. 3O).

Mitochondria in BS and M cells of *S. abrotanoides* had a similar size and structure (~0.4 µm), whereas in the KLCs of *S. deschaseauxiana*, *S. gymnomaschala*, *S. verticillata*, and *R. regelii* they were about 1.3–1.5 times larger compared to the M cells. In KCs and M cells of *S. oppositifolia* the mitochondria were almost identical in size (~0.5 µm).

### Carbon isotope composition (δ^13^C) and CO_2_ compensation point (Г)

Of the species studied biochemically and physiologically here, *S. oppositifolia* had C_4_ δ^13^C values (–13.7 ‰) while the other species had δ^13^C values ranging from –28.8 to –31.5‰, typical for C_3_ plants (Table 2).

**Table 2.** Carbon isotope discrimination (δ^13^C) and CO_2_ compensation point (Г) for a subset of Salsoleae *s.s.* Values with different letters are significantly different according to one-way ANOVA with a *post hoc* Tukey HSD.

Species	Carbon isotope discrimination δ^13^C, ^o^/_oo_	CO_2_ compensation point, *Г*, µmol mol^–1^
*S. abrotanoides*, C_3_	-31.2 ± 0.6 (*n* = 4) a	61.2 ± 0.7 (*n* = 2) a
*R. regelii,* C_3_–C_4_	-31.5 ± 0.3 (*n* = 8) a	36.1 ± 2.2 (*n* = 4) b
*S. deschaseauxiana,* C_3_–C_4_	-29.9 ± 0.3 (*n* = 6) ab	31.9 ± 1.8 (*n* = 4) b
*S. gymnomaschala,* C_3_–C_4_	-28.8 ± 0.3 (*n* = 12) b	31.2 ± 1.0 (*n* = 3) b
*S. divaricata,* C_3_–C_4_	-29.9 ± 0.3 (*n* = 16) ab	33.3 ± 2.5 (*n* = 3) b
*S. verticillata,* C_3_–C_4_	-29.1 ± 0.4 (*n* = 14) b	32.2 ± 2.0 (*n* = 6) b
*S. oppositifolia,* C_4_	-13.0 ± 0.3 (*n* = 6) c	3.7 ± 0.9 (*n* = 4) c

*Г* was measured at 25 °С, 1000 PPFD, and 20% O_2_ in mature leaves of six *Salsola* species and *R. regelii* (Table 2). *Г* values were characteristic of C_4_ species for *S. oppositifolia* (3.7 µmol mol^−1^) and characteristic of C_3_ species for *S. abrotanoides* (61.2 µmol mol^−1^). The *Г* values in the other five species (*R. regelii*, *S. deschaseauxiana*, *S. gymnomaschala*, *S. verticillata*, and *S. divaricata*) were intermediate between C_3_ and C_4_, being about 32 µbar in the four *Salsola* species and 36.2 µmol mol^−1^ in *R. regelii* (Table 2).

### Immunolocalization of GDC

**Fig. 4.**
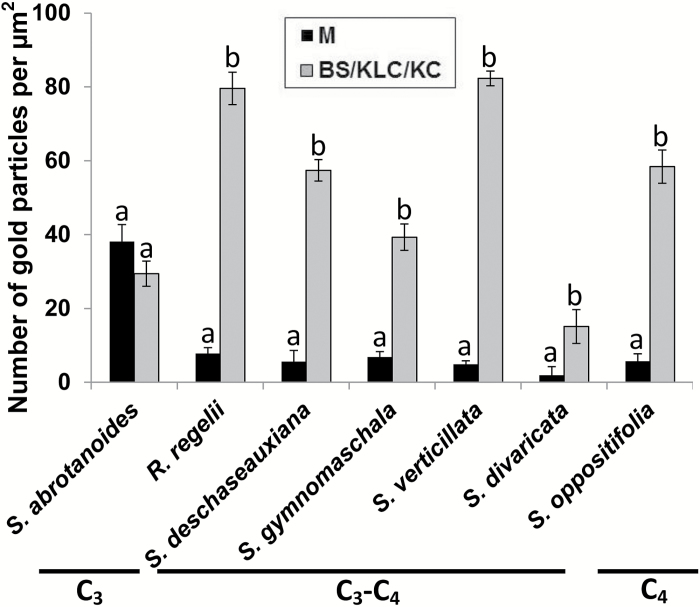
Quantitative data on GDC immunolabeling in mesophyll (M) and bundle sheath (BS)/Kranz-like cells (KLC)/Kranz cells (KC) for a subset of Salsoleae. The background labeling was low and did not exceed 4.0. Different letters indicate significant differences between M and BS/KLC/KC according to Tukey’s HSD (honest significant difference) test.

*In situ* immunogold labeling for GDC using the antibody to the P protein was examined by electron microscopy, and a quantitative analysis was made based on the density of gold particles, in C_3_*S. abrotanoides*, C_3_–C_4_*R. regelii*, *S. deschaseauxiana*, *S. gymnomaschala*, and *S. verticillata*, along with the C_3_–C_4_ species *S. divaricata* and C_4_*S. oppositifolia*. Analysis of the immunolabeling distribution showed that there was no significant difference in density of the gold particles between the mitochondria of M and BS cells in C_3_*S. abrotanoides* (Fig. 4, Supplementary Fig. S2). In contrast, in the C_4_ species *S. oppositifolia* gold particles were selectively localized in KC mitochondria with low labeling in M mitochondria, with a 10-fold difference in their number. In the intermediates *R. regelii*, *S. deschaseauxiana*, *S. gymnomaschala*, and *S. verticillata*, as well as in *S. divaricata*, the number of gold particles was also ~5.8–10 times higher in KLCs compared to M mitochondria (Fig. 4).

### Western blot analysis of key C_4_ enzymes

**Fig. 5.**
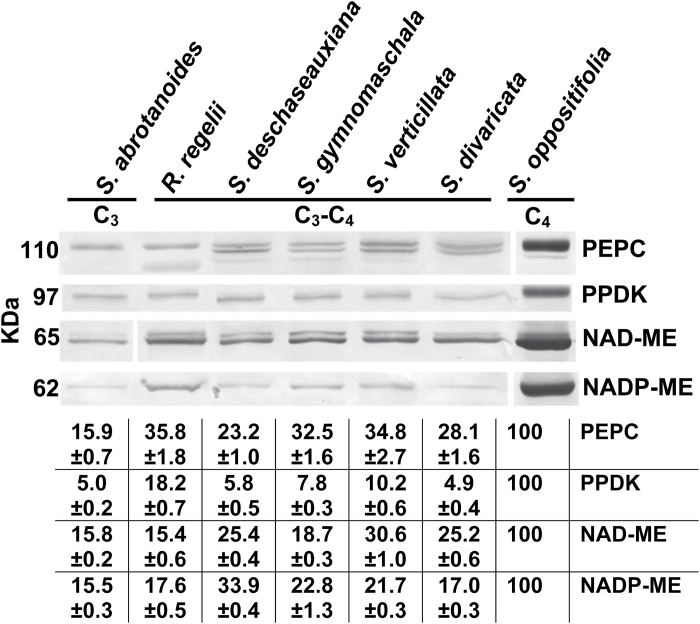
Western blots for C_4_ enzymes from soluble proteins extracted from leaves of six *Salsola s.l.* species, *S. abrotanoides*, *S. deschaseauxiana*, *S. gymnomaschala*, *S. verticillata*, *S. divaricata*, *S. oppositifolia*, and *Rhaphidophyton regelii*. Blots were probed with antibodies raised against PEPC, PPDK, NAD-ME, and NADP-ME: representative western blots are presented showing detection of each protein. The originals were modified for alignment according to species; there were no selective changes in the mass or densities of bands on the membrane. The molecular mass is indicated to the left of the blots. The table gives a quantitative representation of the western blot data in percentage terms, where 100% refers to the level found in leaves of C_4_*S. oppositifolia*.

Immunoblots for the key C_4_ cycle enzymes PEPC, PPDK, NAD-ME, and NADP-ME from total soluble proteins extracted from leaves of the studied species are presented in Fig. 5. The C_4_ species *S. oppositifolia* had very high labelling for the C_4_ pathway enzymes, PEPC and PPDK, and the two decarboxylases, NADP-ME and NAD-ME. Compared to the C_4_ species, the C_3_ species *S. abrotanoides* and the C_3_–C_4_ intermediates *R. regelii*, *S. deschaseauxiana*, *S. gymnomaschala*, *S. verticillata*, and *S. divaricata* had very low labelling for the C_4_ cycle enzyme PPDK and, to varying degrees, less labelling for PEPC, NAD-ME, and NADP-ME.

### Molecular phylogeny of Salsoleae and mapping of key traits

**Fig. 6.**
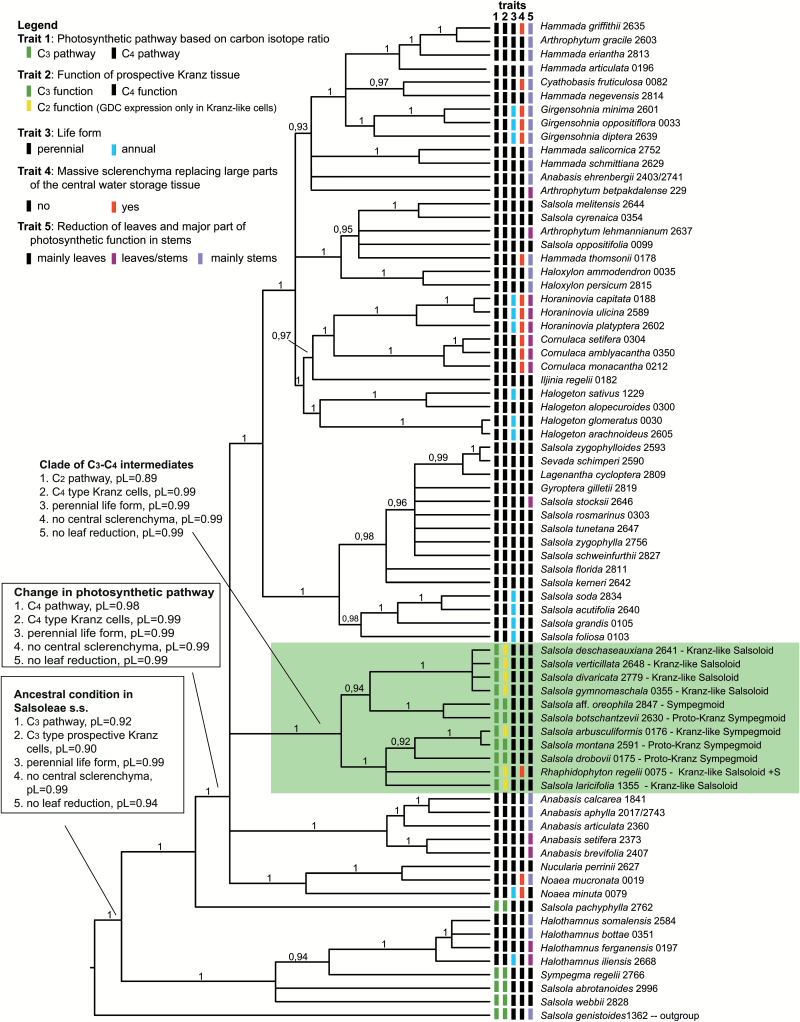
Molecular phylogenetic tree of Salsoleae *s.s.* (Chenopodiaceae) based on four cp markers (*atpB-rbcL* spacer, *ndhF-rpl32* spacer, *trnQ-rps16* spacer, *rpl16* intron) and 74 representative species. The tree was calculated using the program package BEAST (posterior probabilities are shown above branches) and was rooted with *Salsola genistoides*. Character optimization was conducted using Mesquite and ancestral conditions are indicated for selected nodes (pL = proportional likelihood).

The molecular phylogenetic analysis of the chloroplast genome revealed two unambiguous C_4_ lineages in Salsoleae *s.s.*, (1) *Halothamnus*, and (2) *Anabasis* clade + *Noaea* clade + *Haloxylon* clade. Since the *Anabasis* clade, *Noaea* clade, and *Haloxylon* clade formed a polytomy with two C_3_–C_4_ intermediate clades, a higher number of three or four C_4_ origins is possible (Supplementary Fig. S3). When using a narrower outgroup (only *Salsola genistoides*) the two C_3_–C_4_ intermediate clades merged into a monophyletic group that still formed a polytomy with the *Anabasis* clade, *Noaea* clade, and *Haloxylon* clade (Fig. 6). From the crown group age of *Halothamnus* (7.9–1.3 mya), *Anabasis* (10.2–2.6 mya), and *Noaea* (10.3–2.8 mya) it can be assumed that in these lineages C_4_ photosynthesis has been present since the Late Miocene/Early Pliocene. Only for the *Haloxylon* clade can a distinctly older minimum age for the origin of C_4_ photosynthesis of 16.9–6.6 mya be inferred from the molecular dating. In the case of common ancestry of the *Anabasis* clade + *Noaea* clade + *Haloxylon* clade, C_4_ photosynthesis might date back to 19.2–7.6 mya (Supplementary Fig. S3).

The ML character optimization inferred a perennial life form and fully developed leaves without massive central sclerenchyma as the ancestral condition in Salsoleae *s.s.* (Fig. 6). An annual life form evolved at least six times independently in the tribe. A massive central sclerenchyma also evolved repeatedly (Fig. 6). In some cases, this feature was characteristic at the generic level, as in *Girgensohnia*, *Horaninovia*, *Cornulaca*, *Raphidophyton*, and *Noaea*. The reduction or complete loss of a true leaf lamina and a shift of photosynthetic function to the young stems was a common feature in Salsoleae and evolved multiple times in C_4_ lineages of Salsoleae *s.s.*, but also in *S. genistoides*. The occurrence of leafless species was clustered in certain genera, such as *Anabasis*, *Haloxylon*, and *Hammada*, the latter of which seemed to be highly polyphyletic.

Furthermore, the ML character optimization inferred a C_3_ metabolism and C_3_-type BS cells as ancestral in Salsoleae *s.s*. According to the ancestral character state reconstruction, a switch towards C_4_ seems to have already occurred along the branch leading to the large sister group of the C_3_ species *Salsola pachyphylla*, which contains three C_4_ subclades but also one clade of C_3_ and C_3_–C_4_ intermediates (highlighted green in Fig. 6). This clade of C_3_ and C_3_–C_4_ intermediate species did not contain any C_4_ species, and the clade was part of a polytomy of C_4_ clades; thus, there is no indication in the cp tree that the C_3_–C_4_ intermediates represent ancestral states leading towards full C_4_ photosynthesis.

Resolution in the ITS tree was weak in many parts of the tree (Supplementary Fig. S4). Combining cp and ITS data resulted in very low resolution (tree not shown) due to conflicting topologies. Branches that were in conflict between the two data sets (with bootstrap >75) are marked on the ITS tree (Supplementary Fig. S4).

## Discussion

### Evidence for newly identified C_3_–C_4_ species in Salsoleae

Results from gas exchange (*Γ*), compartmentation of GDC between M cells and KLCs, analyses of carbon isotope composition, and analyses of levels of C_4_ enzymes, along with the structure of the respective cells, indicated that four species, *S. deschaseauxiana*, *S. gymnomaschala*, *S. verticillata*, and *R. regelii*, are C_3_–C_4_ intermediates, while *S. abrotanoides* operates C_3_ photosynthesis. Analyses of the carbon isotope composition of these four intermediates as well as the C_3_–C_4_ intermediate *S. divaricata* showed they all have values in the range of those of C_3_ species compared to the C_4_-type value in *S. oppositifolia* (Table 2). However, values for plants grown in growth chambers are more negative (i.e. up to 4–7‰) than samples from natural habitats (Voznesenskaya *et al.*, 2013). The more positive δ^13^C values in the natural habitat may be due to growth under arid conditions limiting CO_2_ diffusion into leaves (Cerling, 1999), or to induction of a partially functional C_4_ cycle. According to our study, among Salsoleae there are at least 19 species with C_3_ isotope values, seven of which are C_3_–C_4_ species (Table 1).

Structural, biochemical, and functional analyses are needed in order to determine whether species having C_3_-type δ^13^C values are C_3_, proto-Kranz, or C_3_–C_4_. An important test is measurement of *Γ*, since values are lower in C_3_–C_4_ than in C_3_ plants, which is indicative of a reduction in photorespiration (Edwards and Ku, 1987). Gas exchange analyses of *S. deschaseauxiana*, *S. gymnomaschala*, *S. verticillata*, and *R. regelii* showed that all these species have *Γ* values that are intermediate between C_4_*S. oppositifolia* and C_3_*S. abrotanoides* (Table 2). Additionally, C_3_–C_4_ intermediates, like C_4_ species, have selective compartmentation of GDC in KLC mitochondria (Rawsthorne *et al.*, 1988; Voznesenskaya *et al.*, 2001, 2013; Sage *et al.*, 2012, 2014), supporting refixation of photorespired CO_2_. Analysis of GDC levels by immunolocalization in these four intermediates indicated selective localization in mitochondria of Kranz-like cells (KLCs), while in the C_3_ species *S. abrotanoides* the density of immunolabeling for GDC was similar in M and BS mitochondria.

Western blot analysis of C_4_ enzymes showed that levels in the C_3_ species *S. abrotanoides* and the C_3_–C_4_ intermediates *S. deschaseauxiana*, *S. gymnomaschala*, *S. verticillata*, and *R. regelii* were very low compared to the C_4_ species *S. oppositifolia*. The levels of PEPC in the four C_3_–C_4_ intermediate species were higher than in the C_3_ species *S. abrotanoides*. However, except for *R. regelii*, levels of PPDK were low and barely detectable in both the C_3_ and intermediate species. *R. regelii* had higher levels of PPDK, but low levels of C_4_ decarboxylases similar to the C_3_ species.

Currrently, the results suggest that all seven known C_3_–C_4_ species of Salsoleae, *R. regelii*, *S. arbusculiformis*, *S. deschaseauxiana*, *S. divaricata*, *S. gymnomaschala*, *S. laricifolia*, and *S. verticillata* (Voznesenskaya *et al.*, 2001, 2013; Wen and Zhang, 2015; this study, Table 1), are Type I, where the reduction of *Г* comes from refixation of photorespired CO_2_ in KLCs with little or no function of a C_4_ cycle (Edwards and Ku, 1987). Whether there is a contribution from a limited C_4_ cycle to photosynthesis in these intermediates could be more directly analyzed by the method of Alonso-Cantabrana and von Caemmerer (2016) via online measurements of photosynthesis and carbon isotope discrimination.

### A model for evolution of C_4_ photosynthesis in Salsoleae based on identified photosynthetic phenotypes

**Fig. 7.**
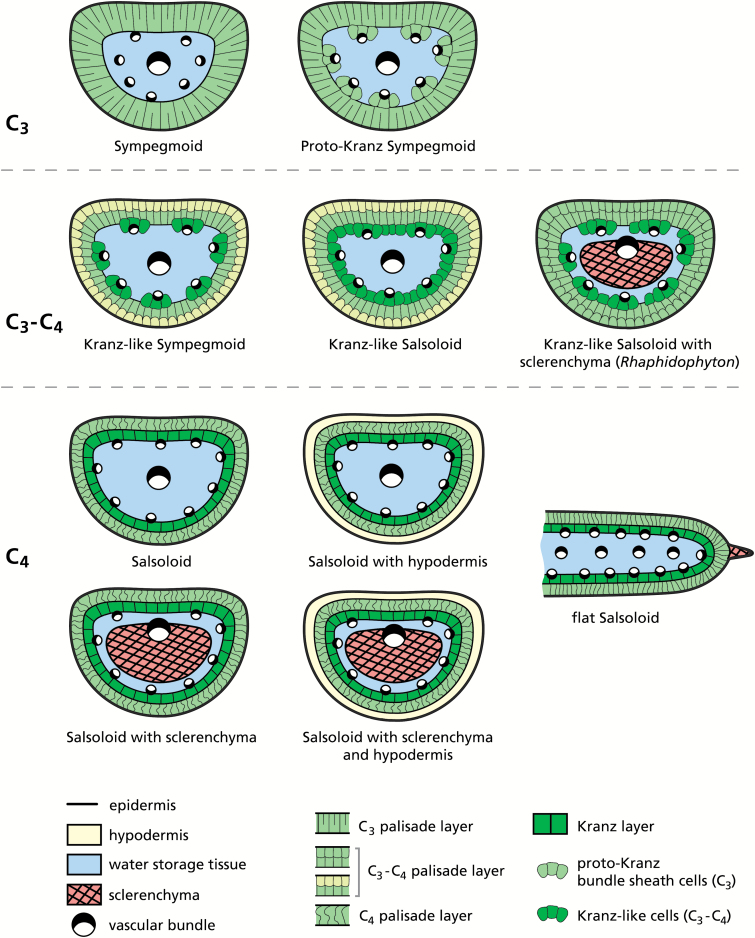
Anatomical schemes of leaf types found in Salsoleae *s.s.*

Of the 77 species of Salsoleae analyzed, without those of the *Kali* clade (Table 1), 24 species were studied for the first time. Our sampling was comprehensive and surpasses Carolin *et al.* (1975) with 43 species and Pyankov *et al.* (2001) with 38 species. Of the 77 species, 19 had C_3_-type carbon isotope composition (consisting of seven C_3_ species, five proposed proto-Kranz species, and seven C_3_–C_4_ intermediates) while 58 were C_4_ species with C_4_-type carbon isotope composition and Salsoloid-type leaf anatomy (Table 1). In Fig. 7 five photosynthetic phenotypes in Salsoleae are described based on the anatomical, ultrastructural, and biochemical analyses of species in the current study together with a few species described by Voznesenskaya *et al.* (2013). In this model, C_4_ is proposed to have evolved structurally and functionally from C_3_ Sympegmoid to Proto-Kranz Sympegmoid to C_3_–C_4_ Kranz-like Sympegmoid to C_3_–C_4_ Kranz-like Salsoloid to C_4_ Salsoloid-type anatomy. There are two subtypes of Kranz-like Salsoloid C_3_–C_4_ intermediates (with or without sclerenchyma) and five anatomical subtypes with Salsoloid type.

#### Non-Kranz anatomy, functionally C_3_

The Sympegmoid leaf type is anatomically and functionally C_3_. It is characterized by usually two well-developed layers of palisade M cells (M1 and M2) and indistinct C_3_-type BS cells around peripheral VBs containing only a few organelles. Species of this type have C_3_ δ^13^C values, C_3_-type *Γ* values, and structural features of M and BS cells characteristic of C_3_ plants (including the occurrence of GDC in both M and BS mitochondria). It is found in *S. abrotanoides* (this study, Figs. 1A, 3, 4), *S. genistoides*, *S. oreophila*, *S. pachyphylla*, *S. webbii* (Carolin *et al.*, 1975; Voznesenskaya, 1976; P’yankov *et al.*, 1997; Pyankov *et al.*, 2001; Voznesenskaya *et al.*, 2013), and *Sympegma regelii* (Wen and Zhang, 2011). Based on anatomical evidence alone, we conclude that *Salsola tianschanica*. belongs to this group, which would then comprise seven species in total (Table 1). An additional trait observed in this group is the comparatively low volume of water storage (WS) tissue and the position of peripheral VBs embedded in the WS tissue rather than at its periphery (Fig. 1A). From known data and the taxonomic literature, in particular the pertinent revisions of section *Coccosalsola* by Botschantzev (1976, 1989), the occurrence of this leaf type in other species is unlikely.

#### Proto-Kranz anatomy, functionally C_3_

Proto-Kranz species have anatomical changes in BS cells that may be the earliest phase of C_4_ evolution, preceding development of the C_2_ cycle (Sage *et al.*, 2014). In Salsoleae, the Proto-Kranz Sympegmoid type only differs from the Sympegmoid type by having distinct cells with chloroplasts and mitochondria arranged preferentially along the inner and the radial walls between peripheral VBs and the chlorenchyma (Fig. 7). Currently this type is only documented in *S. montana* (Voznesenskaya *et al.*, 2013). It has C_3_-like δ^13^C and *Γ* values, and immunolabeling for GDC is similar for M and BS mitochondria. However, based on analysis of leaf anatomy (by light microscopy of fresh leaf or herbarium samples fixed in FAA) there are additional probable candidates for proto-Kranz anatomy among the Central Asian *Salsola* species that have C_3_ δ^13^C values, namely *S. botschantzevii*, *S. drobovii*, *S. junatovii*, and *S. lipschitzii* (Table 1).

#### Kranz-like anatomy, functionally C_3_–C_4_ intermediate

Anatomically there are two types of intermediates in Salsoleae, the Kranz-like Sympegmoid type, and the Kranz-like Salsoloid type (Fig. 7). They resemble C_4_*Salsola* species in having KLCs with numerous organelles in the centripetal position. Both have C_3_-type δ^13^C values, selective localization of GDC in KLC mitochondria, and intermediate *Γ* values indicating functionally C_2_-type species.

The Kranz-like Sympegmoid type is very similar to the aforementioned Sympegmoid forms; but the outer M cells (M1) are distinctly shorter and smaller than the inner M cells (M2) and the KLCs are restricted to the peripheral VBs. This type of structure has so far only been found in *S. arbusculiformis* (Voznesenskaya *et al.*, 2001), and it is suggested to represent the first functional step towards C_4_-type anatomy.

In the Kranz-like Salsoloid type of intermediacy, the M1 appear more like the hypodermal cells in C_4_ species, while still containing more or less numerous chloroplasts. M1 cell size and M/KLC ratio is reduced in comparison with the Sympegmoid types, and the KLCs form a more or less continuous layer (interrupted by crystal-containing idioblasts) around the leaf, as in C_4_*Salsola* species. The KLCs contain chloroplasts and numerous large mitochondria positioned towards the inner cell wall, characteristic of other C_3_–C_4_ intermediate species (Edwards and Ku, 1987; Rawsthorne and Bauwe, 1998; Voznesenskaya *et al.*, 2007, 2010; Muhaidat *et al.*, 2011; Sage *et al.*, 2012, 2014). This type is currently found in six species (Table 1): *S. laricifolia* (Wen and Zhang, 2011, 2015), *S. divaricata* (Voznesenskaya *et al.*, 2013), and *S. deschaseauxiana*, *S. gymnomaschala*, *S. verticillata*, and *R. regelii* (this study, Figs 1 and 7). However, *R. regelii* represents a different subtype by its very strong central sclerenchyma (Fig. 2), which, according to our knowledge, is unique among the C_3_–C_4_ intermediates identified in Salsoloideae.

Of note in the Kranz-like Salsoloid type, considerable variation occurs, mainly in the size, shape and the number of organelles in the M1 cell layer and the arrangement of the KLCs. Sometimes multiple sections within species revealed a certain degree of variation, showing phenotypes more similar to the Kranz-like Sympegmoid type or phenotypes approaching C_4_ plants with typical Salsoloid leaf anatomy. Therefore, more detailed studies are needed to assess the phenotypic plasticity of the functionally intermediate types.

#### Kranz-type anatomy, functionally C_4_

The Salsoloid leaf anatomy in C_4_ lineages of Salsoleae differs substantially from C_4_ eudicots having Atriplicoid-type leaf anatomy with Kranz anatomy around individual veins in flat leaves. Species with Salsoloid-type anatomy are functionally C_4_, with a continuous layer of Kranz cells (KC) around WS tissue and VBs. If the M1 layer of cells is present it occurs as a hypodermis with few or no organelles. There is a further reduction in the M/KC ratio, with organelles in the KCs in a centripetal (or, rarely, in centrifugal) position. In other lineages, as in *Halothamnus*, *Noaea*, *Kali*, *Nanophyton*, and *Climacoptera*, the hypodermis is lacking (Kadereit *et al.*, 2003; Wen and Zhang, 2011). Our data on *S. oppositifolia*, and on several other C_4_ species that had not previously been studied, do not add substantially to the well-known Salsoloid-type anatomy. Together with 40 other species, *S. oppositifolia* displayed the most common Salsoloid type that has a hypodermis and lacks central sclerenchyma; on the other hand only 12 species account for the variant with central sclerenchyma. The Salsoloid type without a hypodermis and without central sclerenchyma was represented in our sampling by four species of *Halothamnus* only. This form is present in many other species of that genus (Kothe-Heinrich, 1993), and in almost all species of *Kali* (Rilke, 1999), while the variant with a central sclerenchyma was seen only in the two *Noaea* species.

### Comparison with the ‘*Flaveria* model’ for C_4_ evolution

The various photosynthetic phenotypes in Salsoleae fit the general model of evolution from C_3_ to proto-Kranz, to intermediates, to Kranz anatomy with a progressive reduction in functional losses due to photorespiration. However, in the ‘*Flaveria* model’ where Kranz anatomy forms around individual veins (Edwards and Ku, 1987; Sage *et al.,* 2012) the structural modifications are very different from the modifications from a Sympegmoid type to a Salsoloid Kranz type where the Kranz anatomy is formed around all veins and WS tissue. In evolution from C_3_ to C_4_ with Kranz anatomy around individual veins, there is increased vein density and size of BS cells around veins as they develop KC features. In contrast, in the Salsoleae the vein density in C_4_ species does not appear to be higher than in the C_3_ species. In addition, the size of the KCs is not significantly increased in the C_4_ species in comparison with their forerunners in C_3_ species (Voznesenskaya *et al.*, 2013). Furthermore, in the Salsoleae model (see fig. 9 in Voznesenskaya *et al.*, 2013) a decrease in the M/KC ratio might also be a precondition, as in grasses (Christin *et al.*, 2013) and dicots (Sage *et al.*, 2012); however, it happens by development of a continuous layer of KCs and reduction in the M1 layer rather than by an increase of veins and BS size.

### Where and when did C_4_ photosynthesis originate in Salsoleae?

The chloroplast gene tree of Salsoloideae resolves five primary clades in the subfamily, namely the *Nanophyton* clade, the Caroxyloneae clade, the *Salsola genistoides* clade, the *Kali* clade, and the Salsoleae *s.s.* clade (Supplementary Fig. S3; Kadereit and Freitag, 2011), with the first four forming the sister group to Salsoleae *s.s.* (Supplementary Fig. S3). Molecular trees based on the nrDNA marker ITS (Supplementary Fig. S4) also reveal these clades but they are contradictory in their positions (for a short discussion on this matter see Supplemetary Fig. S3). According to our results in both data sets only two clearly independent C_4_ lineages in Salsoleae seem likely. The first lineage is *Halothamnus*, with a crown age of 7.9–1.3 mya, and probably plus the *Kali* clade in the ITS data set. Most species of both clades have Salsoloid leaf anatomy and lack a hypodermis (e.g. as in *Traganum*). In the cp tree the monospecific C_3_ genus *Sympegma* is sister to *Halothamnus* while in the ITS tree it is sister to all C_3_–C_4_ intermediates and C_4_ clades in the Salsoleae *sensu lato* (*s.l.*, Supplementary Fig. S4). The *Halothamnus* and *Kali* clades seem to have no close relatives with a C_3_–C_4_ intermediate phenotype. The second C_4_ lineage in Salsoleae *s.s.* probably consists of all other C_4_ species. Since the C_4_ clades of *Anabasis*, *Noaea*, and *Haloxylon* form a polytomy with the C_3_–C_4_ intermediates clade in the cp tree (Fig. 6), one large monophyletic C_4_ clade and a sister-group relationship to the C_3_–C_4_ intermediates remains possible. Unfortunately, the weak resolution of this particular part of the ITS tree within Salsoleae *s.s.* does not support this (Supplementary Fig. S4). The overall similar Salsoloid leaf anatomy with hypodermis (except for the two species of *Noaea*) and NADP-ME biochemistry is in favor of common ancestry of the C_4_ syndrome in this lineage. The predicted age of this large C_4_ lineage in Salsoleae *s.s.* is 19.2–7.6 mya (Supplementary Fig. S3), which is in accordance with the origin of many other C_4_ lineages during the Middle to Late Miocene (Christin *et al.,* 2011).

### Does the C_3_–C_4_ intermediate condition represent an ancestral state to C_4_ in Salsoleae? Is there phylogenetic evidence for a reversion from C_4_ back to C_3_?

The ML optimization suggests that early Salsoleae were shrubs or subshrubs that performed C_3_ photosynthesis in well-developed leaves with a Sympegmoid leaf type (Fig. 6). Along with one C_3_ species (*S. oreophila*), 10 species (consisting of proto-Kranz and C_3_–C_4_ intermediates) in Salsoleae form one (Fig. 6) or two clades (Supplementary Fig. S3) in the cp trees. Lack of resolution in the phylogenetic trees just at the node where these phenotypes and their closest C_4_ relatives arise hampers a reconstruction of these proto-Kranz and C_3_–C_4_ intermediates as ancestral (see also Supplementary Fig. S4). The ML optimization even suggests that the node from which these phenotypes arise was most likely C_4_ (pL=0.98, Fig. 6), which would imply the origin of these C_3_–C_4_ species from C_4_. However, in general a reversion from C_4_ back to C_3_ or intermediate phenotypes seems to be exceedingly rare, if not improbable. Although a few cases have been reported in which C_3_ species or C_3_–C_4_ intermediates are nested in a C_4_ clade, there has also been plausible evidence for a scenario of multiple C_4_ origins (e.g. Ocampo *et al.*, 2013; Bohley *et al.* 2015). In the case of Salsoleae *s.s.*, we assume that a convincing reconstruction is severely hampered by the low number of C_3_–C_4_ intermediate lineages (not by the low number of intermediate species) since all C_3_–C_4_ intermediate species seem to belong to just one lineage.

This C_3_–C_4_ intermediate-rich lineage might, however, be of major interest for future studies. Studying the ecology, physiology, and biochemistry of closely related proto-Kranz and C_3_–C_4_ intermediate species with and without selective localization of GDC might provide further insights into the selective advantage of proto-Kranz anatomy and the C_2_ pathway. So far the selective advantage of displacement of BS organelles towards the centripetal position in proto-Kranz compared to C_3_ species is not clear. Possibly the proto-Kranz state leads to a slight increase in refixation of the CO_2_ generated by GDC in BS cells; however, this could be difficult to detect experimentally (e.g. by measurements of *Γ*). A C_2_ cycle is indeed able to generate distinctly higher CO_2_ levels in leaves (Keerberg *et al.*, 2014) and therefore has an ecophysiological advantage. Since a reversion from intermediate to C_3_ still seems possible in this clade (*S. oreophila*), there is a need for further sampling and deeper resolution within this lineage.

Reduction of leaf lamina combined with photosynthesis being taken over by the green stem cortex evolved multiple times in Salsoleae; however, except for *S. genistoides*, this is only observed in the C_4_ clades. We hypothesize that the higher productivity of the C_4_ cycle in Salsoleae allows for a reduction in the surface area and the amount of photosynthetic tissue, reduction of transpiration, and an increase in water use efficiency. This is obviously advantageous in the extremely dry habitats of the Eurasian deserts and semi-deserts that the Salsoleae have successfully colonized.

## Conclusions

In the model for evolution of C_4_ in Salsoleae, putative C_3_ ancestors have M tissue surrounding the entity of veins with a limited volume of WS tissue; differentiation occurs with development of KLCs next to minor veins, reduction in size of M cells, and ultimately development of an internal layer of KLCs that surrounds all the vascular and WS tissue. Compared to the ‘*Flaveria* model’ for C_4_ development around individual veins, in Salsoleae the proposed biochemical and functional transitions suggest convergence; however, there is obvious divergence in how structural changes were made in C_3_ ancestors to develop Kranz anatomy. In Salsoleae, a number of structural changes that are important in the evolution of C_4_ flat-leaved species are missing: individual KC size often does not increase, but KC volume increases due to the formation of the continuous layer, a decrease of the M/KC ratio occurs mainly due to the reduction of the M1 layer, and the density of venation does not change. These differences might be related to the succulent nature of Salsoleae, with an increase of the volume of WS tissue during the transition from C_3_ to C_3_–C_4_ intermediates and to C_4_ species.

The Salsoleae phylogenies unambiguously reveal all C_3_ species, except for *S. oreophila*, in basal positions, and both the C_4_ species and the C_3_–C_4_ intermediates as derived, but occurring in different clades. Intermediates, proto-Kranz, and one C_3_ species are clustered in one (cp tree) or four (ITS tree) monophyletic groups that might either be sister to a large C_4_ clade or nested within it. In the absence of closely related C_4_ species, their intermediacy cannot be determined as ancestral; although they display logical stepwise, ‘model-conforming’ phenotypes from C_3_ to C_4_ photosynthesis. From a phylogenetic point of view, they may represent an evolutionarily independent solution, enabling the respective species to survive in harsh environments, even in competition with distantly related species of the same tribe possessing full C_4_ photosynthesis.

## Supplementary data

Supplementary data are available at *JXB* online

Table S1. Details of the specimens of Salsoleae and outgroups included in the molecular analyses.

Table S2. Sampling of primary clades of Salsoloideae (Salsoleae, Caroxyloneae, *Nanophyton* clade, *Salsola kali* clade and *Salsola genistoides* clade).

Table S3. Primers, PCR recipes and cycler programs.

Fig. S1. Representative membrane stained with Ponceau S after transfer of proteins to a nitrocellulose membrane and before immunoblotting.

Fig. S2. Electron microscopy of *in situ* immunolocalization of GDC in chlorenchyma cells of *Salsola abrotanoides* (C_3_),

*Rhaphidophyton regelii* (C_3_–C_4_), *S. verticillata* (C_3_–C_4_), and *S. oppositifolia* (C_4_).

Fig. S3. Dated molecular phylogenetic tree of Salsoloideae (Chenopodiaceae) based on four cp markers (*atpB*-*rbcL* spacer, *ndhF*-*rpl32* spacer, *trnQ*-*rps16* spacer, *rpl16* intron).

Fig. S4: ML tree based on ITS sequences of Salsoleae and Caroxyloneae with representatives of Salicornioideae and Suaedoideae as the outgroup.

## Supplementary Material

Supplementary_Figures_S1_S4_Tables_S2_S3

Supplementary_Table_S1

## Abbreviations:

BS: bundle sheath in C_3_ species
Г: CO_2_ compensation point
GDC: glycine decarboxylase
KC: Kranz cell (for C_4_ species)
KLC: Kranz-like cell (for C_3_–C_4_ intermediate phenotypes)
M: mesophyll
ML: maximum likelihood
NAD-ME: NAD-malic enzyme
NADP-ME: NADP-malic enzyme
PEPC: phosphoenolpyruvate carboxylase
PPDK: pyruvate, Pi dikinase
VB: vascular bundle
WS: water storage.
