# Supplementary material for: Molecular phylogeny and forms of photosynthesis in tribe Salsoleae (Chenopodiaceae)
Source: J Exp Bot. 2016 Dec 21;68(2):207–23. doi: 10.1093/jxb/erw432 (PMC5853613; doi:10.1093/jxb/erw432)

Schüssler, C., Freitag, H., Koteyeva, K., Schmidt, D., Edwards, G., Voznesenskaya, E., Kadereit, G.: Molecular phylogeny and forms of photosynthesis in tribe Salsoleae (Chenopodiaceae)

Supplementary Table S2: Sampling of primary clades of Salsoloideae (Salsoleae, Caroxyloneae, *Nanophyton* clade, *Salsola kali* clade and *Salsola genistoides* clade).

**Taxa**	**Spp.**	**Species sampled (lab code)**
**Salsoleae s.s. clade; c. 19 gen., c. 160 spp. (sampling comprises all genera and 75 species)**		
Anabasis L.	29	*A. aphylla* L. (2017, 2743), *A. articulata* (Forssk.) Moq. (2360), *A. brevifolia* C.A. Mey. (2407), *A. calcarea* (Charif & Aellen) Bokhari & Wendelbo (1841), *A. ehrenbergii* Schweinf. ex Boiss. (2403, 2741), *A. setifera* Moq. (2373)
Arthrophytum Schrenk	9	*Ar. betpakdalense* Korovin (0229), *Ar. gracile* Aellen (2603), *Ar. lehmannianum* Bunge (2637)
Cornulaca Delile	8	*C. amblyacantha* Bunge (0350), *C. monacantha* Delile (0212), *C. setifera* (DC.) Moq. (0304)
Cyathobasis Aellen	1	*Cy. fruticulosa* (Bunge) Aellen (0082)
Girgensohnia Bunge	5	*G. diptera* Bunge (2639), *G. minima* Korovin (2601), *G. oppositiflora* (Pall.) Fenzl (0033)
Gyroptera Botsch.	2	*G. gilletii* Botsch. (2819)
Halogeton C.A. Mey.	5	*Hg. alopecuroides* (Delile) Moq. (0300), *Hg. arachnoideus* Moq. (2605), *Hg. glomeratus* (M. Bieb.) C.A. Mey. (0030), *Hg. sativus* (L.) Moq. (1229)
Halothamnus Jaub. & Spach	21	*Ht. bottae* Jaub. & Spach (0351), *Ht. ferganensis* Botsch. (0197), *Ht. iliensis* (Lipsky) Botsch. (2668), *Ht. somalensis* (N.E. Br.) Botsch. (2584)
Haloxylon Bunge	2	*Hx. ammodendron* (C.A. Mey.) Bunge (0035), *Hx. persicum* Bunge ex Boiss. (2815)
Hammada Iljin	16	*Ha. articulata* (Moq.) O. Bolos & Vigo (0196), *Ha. eriantha* Botsch. (2813), *Ha.griffithii* (Moq.)Ilji (2635), *Ha. negevensis* Iljin & Zohary (2814), *Ha. salicornica* (Moq.) Iljin (2752), *Ha. schmittiana* (Pomel) Botsch. (2629), *Ha. scoparia* (Pomel) Iljin (2633), *Ha. thomsonii* (Bunge) Iljin (0178)
Horaninovia Fisch. & C.A. Mey.	7	*Ho. capitata* Sukhor. (0188), *Ho. platyptera* Charif & Aellen (2602), *Ho. ulicina* Fisch. & C.A. Mey. (2589)
Iljinia Korovin	1	*I. regelii* (Bunge) Korovin (0182)
Lagenantha Chiov.	1	*L. cycloptera* (Stapf) M.G. Gilbert & Friis (2809)

Noaea Moq.	3	*N. minuta* Boiss. & Balansa. (0079), *N. mucronata* (Forssk.) Asch. & Schweinf. (0019),
Nucularia Batt.	1	*Nu. perrinii* Batt. (2627)
Rhaphidophyton Iljin	1	*R. regelii* (Bunge) Iljin (0075)
Salsola L.	> 40	*S. abrotanoides* Bunge (2996), *S. acutifolia* (Bunge) Botsch. (2640), *S. annua* (Bunge) Akhani (2832), *S. arbusculiformis* Drobow (176), *S. botschantzevii* Kurbanov (2630), *S. cyrenaica* (Maire & Weiller) Brullo (354), *S. deschaseauxiana* Litard. & Maire (2641, 2758), *S. divaricata* Masson ex Link (2779, 2829), *S. drobovii* Botsch. (0175), *S. florida* (M. Bieb.) Poir. (2811), *S. foliosa* (L.) Schrad. (0103), *S. grandis* Freitag, Vural & N. Adıgüzel (0105), *S. gymnomaschala* Maire (0355), *S. kerneri* (Woł.) Botsch. (2642), *S. laricifolia* Turcz. ex Litv. (1355), *S. melitensis* Botsch. (2644), *S. montana* Litv. (2591), *S. oppositifolia* Desf. (0099), *S. oreophila* Botsch. (2847), *S. pachyphylla* Botsch. (2632, 2762), *S. rosmarinus* (Ehrenb. ex Boiss.) Akhani (0303), *S. schweinfurthii* Solms-Laub. (2827), *S. soda* L. (2834), *S. stocksii* Boiss. (2646), *S. tunetana* Brullo (2647), *S. verticillata* Schousb. (2648), *S. webbii* Moq. (2828), *S. zygophylla* Batt. & Trab. (2756), *S. zygophylloides* (Aellen & Townsend) Akhani (2593)
Sevada Moq.	1	*Se. schimperi* Moq. (2590)
Sympegma Bunge	1	*Sy. regelii* Bunge (2766, 383a)
**Other primary clades of Salsoloideae**		
***Salsola kali* clade (=*Kali* clade), 4 gen., c. 22 spp.**		
Kali collina (Pall.) Akhani & E.H. Roalson (0207, 2592), *K. komarovii* (Iljin) Akhani & E.H. Roalson (2649), *K. monoptera* (Bunge) Lomon. (177), *Traganopsis glomerata* Maire & Wilczek (2609), *Xylosalsola arbuscula* (Pall.) Tzvelev. (2586), *X. paletzkiana* (Litv.) Akhani & E. H. Roalson (2606)		
***Nanophyton* clade, 1 gen., c. 12 spp.**		
Nanophyton erinaceum Bunge (1346), *Nanophyton grubovii* U.P. Pratov (0347)		
***Salsola genistoides* clade; gen. nov., 1 spp.**		
Salsola genistoides Juss. ex Poir. (1155, 1362)		
**Caroxyloneae clade, 11 gen., c. 145 spp.**		
Caroxylon spinescens (Moq.) Akhani & Roalson (2749, 2597), *Caroxylon imbricatum* (Forssk.) Moq. (2703), *Gamanthus gamocarpus* Bunge (2588), *Kaviria rubescens* (Franch.) Akhani (0200, 2751), *Caroxylon omanensis* (Boulos) Freitag & G. Kadereit)* (2750), *Petrosimonia triandra* (Pall.) Rech. (2578)		

Names according to Akhani *et al.* (2007), Kadereit and Freitag (2011) and Kadereit *et al.* (2012). Full references see main text. For voucher information and Genbank accession numbers see Appendix Table S1.

)* *Caroxylon omanensis* (Boulos) Freitag & G. Kadereit, comb. nov. Basionym: *Salsola omanensis* Boulos, Kew Bull. 46: 297. 1991. The species is recombined here because according to the protologue, the figure 51A given in Miller AG, Cope TA. 1996. Flora of the Arabian Peninsula and Socotra. Vol. 1. Edinburgh University Press, the rich material recollected near the type locality, and its sequences it clearly belongs to *Caroxylon.*

Supplementary Table S3: Primers, PCR recipes and cycler programs

Marker	Primer sequences and references	PCR recipe (µl)	Cycler program
ITS	ITSA 5‘-GGA AGG AGA AGT CGT AAC AAG G-3‘; ITSC 5‘-GCA ATT CAC ACC AAG TAT CGC-3‘; ITSD 5’-CTC TCG GCA ACG GAT ATC TCG-3‘ (all three of them Blattner 1999); ITS4 5’-TCC TCC GCT TAT TGA TAT GC-3’ (White *et al.* 1990)	ddH_2O 17.33, $MgCl_2$ (25 mM) 0.75, buffer 2.5, DMSO 1.0, dNTPs (10 mM) 0.25, taq polymerase 0.17, F primer (50 µM) 0.5, R primer (50µM) 0.5, DNA template 2	94°C for 1 min, 35 cycles of [94°C for 30 s, 52°C for 50 s. 72°C for 1 min], 94°C for 30 s, 52°C for 1 min 12s, 72°C for 8 min
rpl16 intron	rpl16 F71 5‘-GCT ATG CTT AGT GTG TGA CTC GTT G-3' (Shaw *et al.* 2005); rpl16 intern R2 5‘-CAC GGG TTC CAT CGT TCC CAT CGC-3‘; rpl16 intern F 5‘-GCG ATG GGA ACG ATG GAA CCC GTG-3‘; rpl16 R1516 5‘-CCC TTC ATT CTT CCT CTA TGT TG-3‘ (Shaw *et al.* 2005)	ddH_2O 18.8, $MgCl_2$ (25 mM) 1, buffer 2.5, BSA (10mg/ml) 0.25, dNTPs (10 mM) 0.25, taq polymerase 0.2, F primer (50 µM) 0.5, R primer (50µM) 0.5, DNA template 1	80°C for 5 min, 35 cycles of [95°C for 1 min, 50°C for 1 min, 65°C* for 4 min], 65°C for 5 min<br>* increasing in 0.3°C steps
trnQ-rps16 spacer	trnQ$^{(UUG)}$ 5‘-GCG TGG CCA AGY GGT AAG GC-3‘ (Shaw *et al.* 2007); trnQ intern R 5‘-GGA ATT CAA CTT CCT GGA ATC TAG AAC CCC-3‘; trnQ intern F 5‘-GGG GTT CTA GAT TCC AGG AAG TTG AAT TCC-3‘; rps16x1 5‘-GTT GCT TTY TAC CAC ATC GTT T-3‘ (Shaw *et al.* 2007)		
ndhF-rpl32 spacer	ndhF 5‘-GAA AGG TAT KAT CCA YGM ATA TT -3‘(Shaw *et al.* 2007); ndhF-RM1r 5’-GTA TTC ACG TTC ACT AAC AAA TTG G-3’; ndhF-RM1f 5‘-CCA ATT TGT TAG TGA ACG TGA ATA C-3‘; ndhF-RM2r 5‘-CAC ATT GAA AAC TTT AAG AAC TCA ACC-3‘; ndhF-RM2f 5‘-GGT TGA GTT CTT AAA GTT TTC AAT GTG-3‘; rpl32 R 5‘-CCA ATA TCC CTT YYT TTT CCA A -3‘ (Shaw *et al.* 2007)		
atpB-rbcL spacer	atpB-rbcL-spacer F 5‘-GAA GTA GTA GGA TTG ATT CTC-3‘ (Xu *et al.* 2000); atpB-rbcL spacer F2 5‘-GGA GTT AGC AAT CCA TTT TGT TGG-3‘; atpB-rbcL intern R2 5‘-CGA AAT TTA CCC TTG ACA GCG G-3‘; atpB-rbcL intern F2 5’-CCG CTG TCA AGG GTA AAT TTC G-3‘; atpB-rbcL-spacer R 5‘-CAA CAC TTG CTT TAG TCT CTG-3‘ (Xu *et al.* 2000)	ddH_2O 19.2, $MgCl_2$ (25 mM) 0.6, buffer 2.5, BSA (10mg/ml) 0.25, dNTPs (10 mM) 0.25, taq polymerase 0.2, F primer (50 µM) 0.5, R primer (50 µM) 0.5, DNA template 1	94°C for 1 min, 35 cycles of [94°C for 30 s, 52°C for 50 s, 72°C for 1 min], 94°C for 30 s, 52°C for 1 min 12 s, 72°C for 8 min

References:

Blattner, F. (1999). Direct amplification of the entire ITS region from poorly preserved plant material using recombinant PCR. BioTechniques 27(6):1180-1186.

Shaw, J., Lickey, E. B., Beck, J. T., Farmer, S. B., Liu, W., Miller, J., Siripun, K. C., Winder, C. T., Schilling, E. E. und Small R. L. (2005). The tortoise and the hare II: relative utility of 21 noncoding chloroplast DNA sequences for phylogenetic analysis. American Journal of Botany, 92 (1): 142–166.

Shaw, J., Lickey, E. B., Schilling, E. E. und Small, R. L. (2007). Comparison of whole chloroplast genome sequences to choose noncoding regions for phylogenetic studies in angiosperms: the tortoise and the hare III. American Journal of Botany, 94 (3): 275-288.

White, T. J., Brims, T., Lee, S., and Taylor, J. (1990). Amplification and direct sequencing of fungal ribosomal RNA genes for phyloge- netics. In “PCR Protocols: A Guide to Methods and Applications” (M. Innis, D. Gelfand, J. Sninsky, and T. White, Eds.), pp. 315- 322, Academic Press, San Diego.

Xu, D.H., Abe, J., Sakai, M., Kanazawa, A. & Shimamoto, Y. 2000. Sequence variation of non-coding regions of chloroplast DNA of soybean and related wild species and its implications for the evolution of different chloroplast haplotypes. Theor. Appl. Genet. 101: 724--732. DOI: 10.1007/s001220051537

Schüssler, C., Freitag, H., Koteyeva, K., Schmidt, D., Edwards, G., Voznesenskaya, E., Kadereit, G.: Molecular phylogeny and forms of photosynthesis in tribe Salsoleae (Chenopodiaceae)

Figure S1: Representative membranes stained with Ponceau S after proteins (20 µg per lane) were transferred to nitrocellulose membrane and before immunoblotting. The loading controls show clear banding patterns for the soluble protein extracts with no apparent degradation by proteases. The higher intensity staining at 55 Kda was confirmed by western blot analysis to be ribulose-1,5-bisphosphate carboxylase large subunit (RbcL). The intermediates and the one C_4 species have much lower levels of RbcL in the soluble protein extracts than the one C_3 species. This may represent the in vivo levels of the individuals which were analyzed, although there is some potential for some loss of Rubisco due to binding to thylakoid membranes following extraction (Makino and Osmond, 1991).

Makino A, Osmond B. 1991. Solubilization of ribulose-1,5-bisphosphate carboxylase from the membrane fraction of pea leaves. Photosynthesis Research **29**: 79-85.

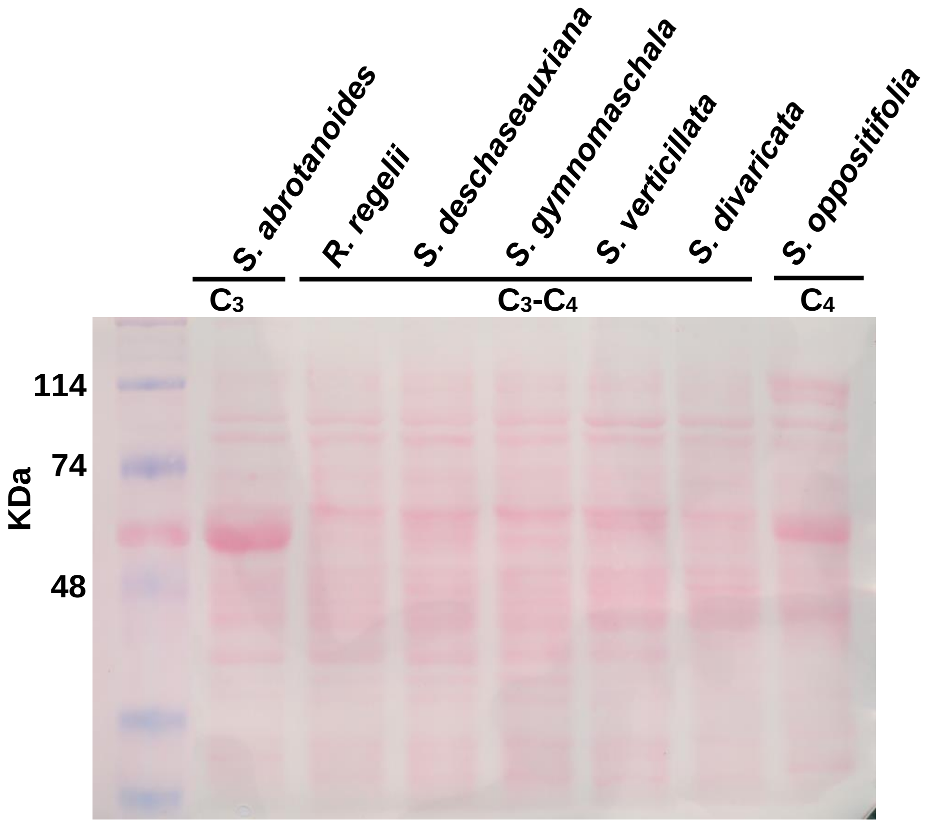

Figure S2: Electron microscopy of *in situ* immunolocalization of glycine decarboxylase (GDC) in chlorenchyma cells of *Salsola abrotanoides* (C_3), *Rhaphidophyton regelii* (C_3–C_4), *S. verticillata* (C_3–C_4) and *S. oppositifolia* (C_4). Gold particles in M and BS/KLC/KC cells in (*A*, *B*) *Salsola abrotanoides*, (*C*, *D*) *Rhaphidophyton regelii*, (*E*, *F*) *S. verticillata* and *(G, H) S. oppositifolia*. Scale bars=0.5 µm. c, chloroplast; m, mitochondria.

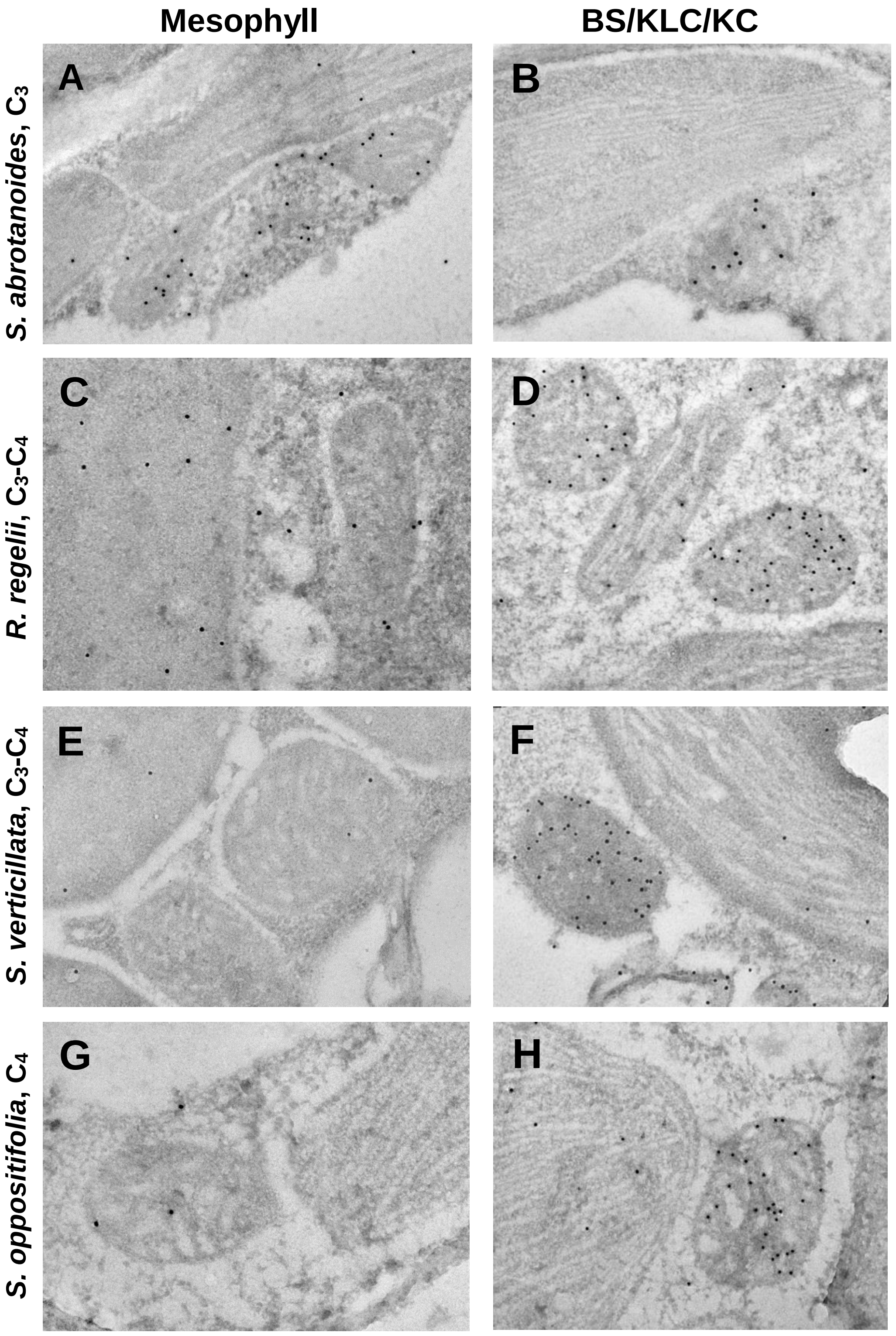

Figure S3: Molecular dated phylogenetic tree of Salsoloideae (Chenopodiaceae) based on four cp marker (atpB-rbcL spacer, ndhF-rpl32 spacer, trnQ-rps16 spacer, rpl16 intron) and Bayesian inference.

The chloroplast gene tree of Salsoloideae resolves five primary clades in the subfamily, namely Nanophyton clade, Caroxyloneae clade, Salsola genistoides clade, Kali clade, and Salsoleae s.s. clade (see also Kadereit & Freitag 2011), with the former four forming the sister group to Salsoleae s.s.
Molecular trees based on the nrDNA marker ITS also reveal these clades but they contradict in their position. In the ITS trees the Kali clade falls into the Salsoleae s.s. and the Nanophyton clade falls into the Caroxyloneae clade (Akhani et al. 2007; Voznesenskaya et al. 2013; Fig. S2). This topological conflict points to ancient hybridization events in the Salsoloideae and hampers the reconstruction of C4 evolution in the subfamily which is extremely rich in C4 species, but it also contains a considerable number of C3 species. The Nanophyton clade, Kali clade and Caroxyloneae clade consist entirely of C4 species, while the phylogenetically more isolated species, Salsola genistoides, is a C3 species and the species-rich Salsoleae s.s. also contain several C3 species. Of great importance for the reconstruction of the C4 origin in Salsoloideae is the position of C3 species such as Salsola genistoides, S. webbii and S. abrotanoides as well as S. pachyphylla. Cp and nuclear data agree that Caroxyloneae and Nanophyton stand outside Salsoleae. These two clades likely represent at least one independent origin of C4 photosynthesis in Salsoloideae since the Caroxyloneae differ in their biochemical subtype (NAD-ME) from Salsoleae s.s. and the Kali clade (both NADP-ME, Kadereit et al. 2003 and refs. therein). The C4 subtype of Nanophyton was not studied biochemically but structurally belongs to the NADP-ME group of species (Voznesenskaya, unpublished data). According to the current molecular dating of Chenopodiaceae, the Caroxyloneae and the Kali clade likely represent the oldest C4 lineages of the family dating back to the early Oligocene (Kadereit et al. 2012) while C4 lineages in Salsoleae s.s. are found to be distinctly younger dating back to the Middle to Late Miocene.

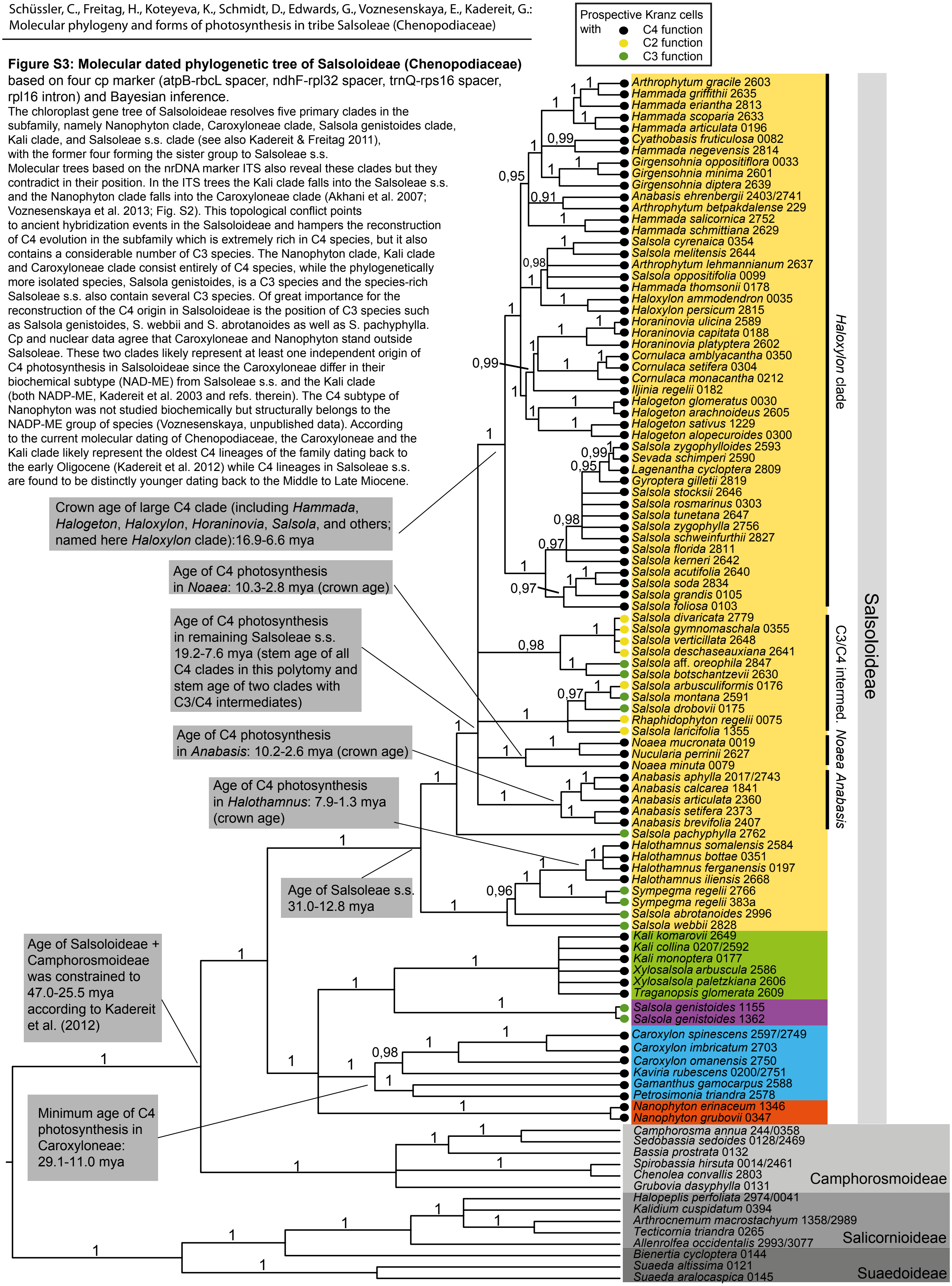

Schüssler, C., Freitag, H., Koteyeva, K., Schmidt, D., Edwards, G., Voznesenskaya, E., Kadereit, G.:
Molecular phylogeny and forms of photosynthesis in tribe Salsoleae (Chenopodiaceae)

Suppl. Figure S4

ML tree based on ITS sequences of Salsoleae and Caroxyloneae with representatives of Salicornoideae and Suaedoideae as outgroup. ML bootstrap values above branches. Branches marked with * are in conflict with the tree based on cp data.

C3 function

C2 function (GDC only expressed in Kranz-like cells)

C4 function

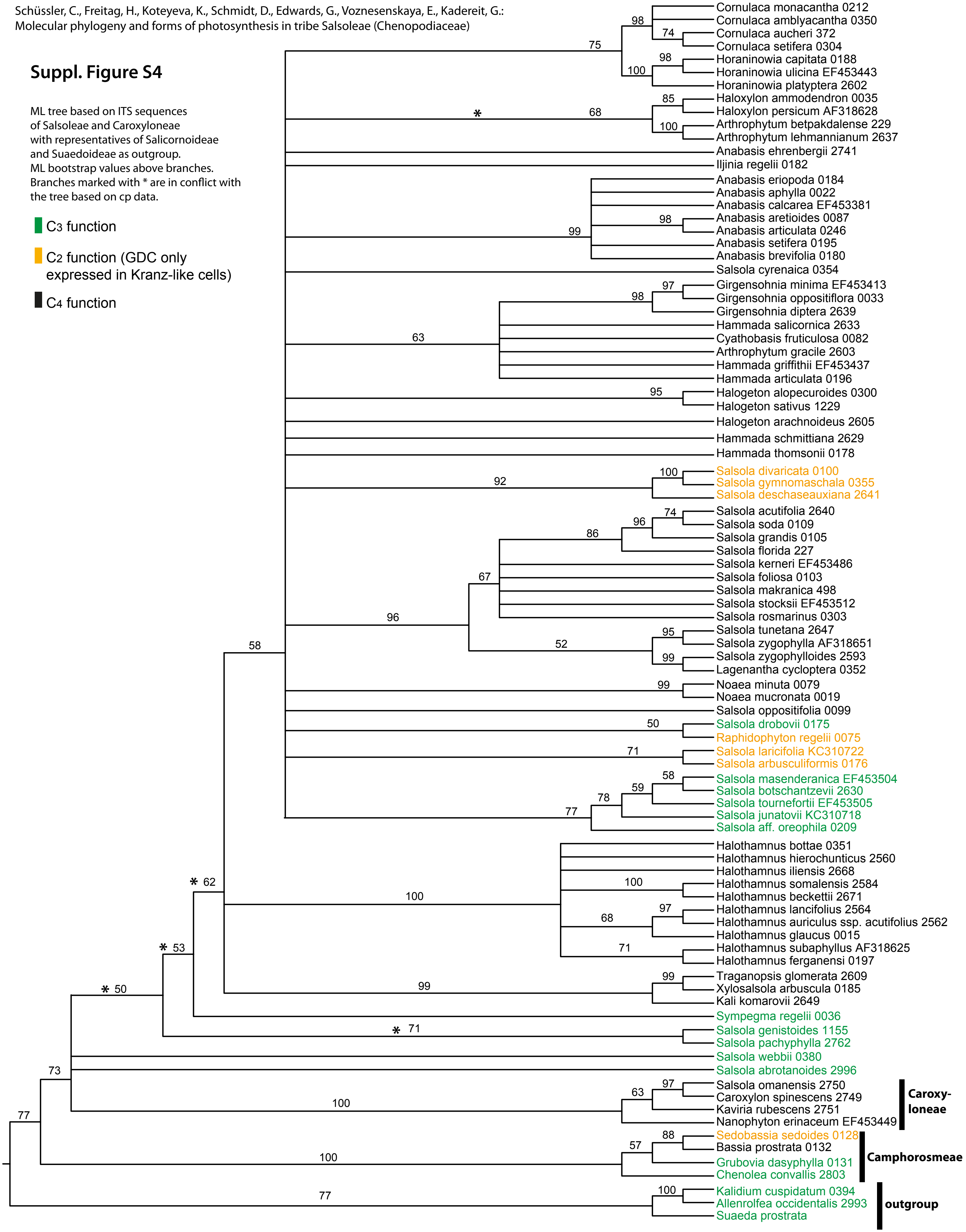

Supplement: Supplementary_Figures_S1_S4_Tables_S2_S3 [file erw432_suppl_supplementary_figures_s1_s4_tables_s2_s3.pdf]
